# A novel double-ribonuclease toxin-antitoxin system linked to the stress response and survival of *Acidovorax citrulli*

**DOI:** 10.1128/spectrum.02169-23

**Published:** 2023-10-11

**Authors:** Xudong Wang, Yumin Kan, Kaihong Bai, Xiaoli Xu, Xing Chen, Chengxuan Yu, Jia Shi, Na Jiang, Jianqiang Li, Laixin Luo

**Affiliations:** 1 Department of Plant Pathology, College of Plant Protection, China Agricultural University, Beijing, China; 2 Key Laboratory of Surveillance and Management for Plant Quarantine Pests, Ministry of Agriculture and Rural Affairs, Beijing, China; 3 Beijing Key Laboratory of Seed Disease Testing and Control, Beijing, China; 4 Department of Biochemistry and Cell Biology, State University of New York, Stony Brook, New York, USA; 5 School of Life Sciences, Zhengzhou University, Zhengzhou, Henan, China; USDA - San Joaquin Valley Agricultural Sciences Center, Parlier, California, USA

**Keywords:** *Acidovorax citrulli*, toxin-antitoxin, ribonuclease, biofilm, stress response

## Abstract

Toxin-antitoxin (TA) systems are widespread among prokaryotes and can play an important role in the maintenance of genetic elements and pathogen virulence. Their roles in bacterial stress response and antimicrobial persistence currently remain highly controversial. The current study investigated a novel TA system (Aave_1720-Aave_1719) from the phytopathogenic bacterium *Acidovorax citrulli*, which had characteristics of both type II and type V TA systems. Heterologous expression in *Escherichia coli* confirmed that *Aave_1720* functions as a toxin gene, while *Aave_1719* acts as antitoxin gene. The Aave_1720 toxin functions as a ribonuclease, and antitoxin Aave_1719 binds directly to Aave_1720 to inhibit its ribonuclease activity, which is typical of type II TA systems. Interestingly, Aave_1719 itself was found to exhibit ribonuclease activity with a preference for targeting *Aave_1720* mRNA, which indicates that it can also neutralize the Aave_1720 toxin in a manner characteristic of type V TA systems. Expression analysis revealed that the *Aave_1719* gene is upregulated in response to sodium hypochlorite stress and that deletion of the *Aave_1720* gene individually or in combination with *Aave_1719* affected biofilm formation, as well as the survival of *A. citrulli* in response to sodium hypochlorite stress. Taken together, these findings expand our understanding of TA systems revealing a novel mechanism of toxin neutralization that combines aspects of both type II and type V TA systems.

**IMPORTANCE:**

Bacterial fruit blotch (BFB), which is caused by the seed-borne bacterium *Acidovorax citrulli*, is a devastating disease affecting cucurbit crops throughout the world. Although seed fermentation and treatment with disinfectants can provide effective management of BFB, they cannot completely guarantee pathogen-free seedstock, which suggests that *A. citrulli* is a highly stress-resistant pathogen. Toxin-antitoxin (TA) systems are common among a diverse range of bacteria and have been reported to play a role in bacterial stress response. However, there is currently much debate about the relationship between TA systems and stress response in bacteria. The current study characterized a novel TA system (Aave_1720-Aave_1719) from *A. citrulli* that affects both biofilm formation and survival in response to sodium hypochlorite stress. The mechanism of neutralization differed from typical TA systems as two separate mechanisms were associated with the antitoxin, which exhibited characteristics of both type II and type V TA systems. The Aave_1720-Aave_1719 system described here also constitutes the first known report of a double-ribonuclease TA system in bacteria, which expands our understanding of the range of regulatory mechanisms utilized by bacterial TA systems, providing new insight into the survival of *A. citrulli* in response to stress.

## INTRODUCTION

*Acidovorax citrulli* is a plant pathogenic bacterium that causes bacterial fruit blotch (BFB) in cucurbits (1). Although first reported in 1965, this pathogen was not given much attention until the BFB outbreak in watermelon cultivated in the Mariana islands during the 1987 growing season (2, 3). Today, BFB is recognized as an economically important disease of cucurbits throughout the world (4). Under favorable environmental conditions, BFB can spread rapidly causing yield losses of up to 80%. The *A. citrulli* bacterium is primarily a seed-borne pathogen that is transmitted via contaminated seedstock. In the absence of commercial cucurbit varieties with durable resistance to *A. citrulli*, disease management relies primarily on the planting of non-infected seeds, which can be produced by seed fermentation, or treatment with disinfectants such as HCl and NaClO (5). Heowever, these methods are not completely effective, and BFB outbreaks can still occur.

In 2007, the genome (GenBank CP000512.1) of the *A. citrulli* model strain, AAC00-1, was fully sequenced, which facilitated much more convenient investigation of gene function. Although there have been many recent studies regarding the pathogenicity and virulence factors of *A. citrulli*, little attention has been given to its stress response, or the potential for toxin-antitoxin (TA) systems to influence this process. Such systems are widespread among Bacteria and Archaea species and generally consist of two tightly linked genes (6). They were first described in the much studied bacterial species, *Escherichia coli*, in which TA systems were found to ensure the inheritance of plasmids by killing cells that lost the plasmid during cell division (7). However, with the advent of genome sequencing, TA systems were found to be a widespread phenomenon that also appeared in chromosomal DNA (8) and were associated with many biological processes, including biofilm formation (9), the virulence of pathogenic bacteria (10), the inhibition of bacteriophages by abortive infection (11), and the bacterial stress response (12). However, there is ongoing controversy surrounding the role of TA systems in bacterial stress response and antimicrobial persistence.

In general, TA systems are encoded by two separate components: a stable protein toxin (except in type VIII systems where the toxin is RNA in nature) and a cognate unstable antitoxin, which can be either protein or RNA in nature (13). It is generally considered that in favorable conditions, the antitoxin completely blocks the activity of the toxin, but under stress, the antitoxin is prone to degradation and the toxin is activated (14). Different toxins target a wide range of cellular processes including DNA replication, translation, RNA degradation, and the maintenance of cell envelope integrity, as well as by the induction of metabolic stress (13). Similarly, there are numerous mechanisms by which the antitoxin can interact with the toxin in order to neutralize its effect. Indeed, this diversity in mechanisms has led to the classification system used to categorize eight different types of TA system (13 – 15). Type I antitoxins are small noncoding RNA molecules that bind to the mRNA of the toxin resulting in its degradation or the inhibition of ribosome binding, which prevents translation of the toxin protein (16 – 18). The type II TA system is the most common and best understood of all chromosomal TA systems and differs from type I in that the antitoxin itself is a protein and neutralizes the toxin by binding to it directly. Another feature of the type II TA system is that the toxin/antitoxin complex can autoregulate its own expression by binding to its coding operon (19, 20). Type III antitoxins also bind to the toxin directly, but in this case. the antitoxin is RNA in nature (21, 22). However, the type IV TA system differs from the other three in that the antitoxin protein binds to the target of toxin, which protects it from the activity of the toxin (23, 24). Meanwhile, the type V TA system employs another strategy in which the antitoxin protein has RNase activity that directly targets and degrades the mRNA of the toxin component (25). The type VI antitoxins are somewhat analogous, but in this case, the protein antitoxin binds to the toxin itself and targets it for degradation by the ClpXP protease (26). The type VII antitoxins are also protein in nature and exert their effect not by binding to the toxin directly but by performing post-translation modifications that deactivate it (27). In type VIII TA systems, which are the only category to employ an RNA-based toxin, the RNA antitoxin functions either by binding to the toxin RNA directly to form an inactive duplex or by interacting with the CRISPR-Cas system in a way that prevents transcription of the toxin RNA (28, 29).

The majority of research regarding bacterial TA systems has focused on mammalian pathogens, such as *E. coli*, *Mycobacterium tuberculosis*, *Staphylococcus aureus,* and *Pseudomonas aeruginosa*, for example, the BrnTA system of the zoonotic pathogen *Brucella abortus*, which is a typical type II TA system consisting of a ribonuclease toxin, BrnT, in conjunction with the BrnA antitoxin, which neutralizes its toxicity by forming a 2:2 tetrameric complex. It has also been shown that both BrnA alone and the BrnTA complex can bind to the promoter of the *BrnTA* operon to autoregulate its transcription, while transcription of the toxin itself is strongly induced by multiple stressors including the antibiotic chloramphenicol, hydrogen peroxide, and low pH (30). Although TA systems are also abundant among plant-pathogenic bacteria, they have drawn much less attention (31). To date, at least 13 TA systems have been described in plant pathogenic bacteria, including the ToxI-ToxN in *Erwinia carotovora* subsp. *atroseptica* (21), PemI-PemK, DinJ-RelE and MqsR-YgiT in *Xylella fastidiosa* (32, 33), StbD-StbE in *Erwinia pyrifoliae* (34), AvrRxo1-Arc1 in *Xanthomonas oryzae* pv. *oryzicola* (35), Hok-Sok in *Erwinia amylovora* (36), EcnA-EcnB in *Xanthomonas citri* subsp. *citri* (37), CcdA-CcdB, Phd-Doc and DhiT-DhiA in *Dickeya dadantii* (38), and MazE-MazF in *Agrobacterium tumefaciens* (39).

Up to now, only one TA system has been reported in *A. citrulli*, the VapBC system, which is a co-transcribed type II TA system comprised of the ribonuclease toxin VapC and the VapB antitoxin (40). However, searches of the type II toxin-antitoxin database (TADB 2.0) (41) suggest that *A. citrulli* might have at least one additional TA system encoded by the *Aave_1720-Aave_1719* operon. The current study was initiated in order to characterize this putative *A. citrulli* TA system experimentally using a series of microbiological, molecular, and biochemical analyses.

## MATERIALS AND METHODS

### Bacterial strains, plasmids, and growth conditions

The bacterial strains and plasmids used in the current study have been listed in TableS4. The experimental *E. coli* strains were grown in lysogeny broth (LB) at 37°C with shaking (200 rpm) or on lysogeny broth containing 1.8% (wt/vol) agar (LBA) and supplemented with 0.2% arabinose or 0.2% glucose when necessary, while the *A. citrulli* strain AAC00-1 was grown in LB at 28°C with shaking (150 rpm) or on LBA. The antibiotics used for selection in *E. coli* were added to either solid or liquid media as follows: ampicillin (50 µg mL^−1^) for the maintenance of the pBAD plasmids and kanamycin (50 µg mL^−1^) for the pET28a and pK18*mobsacB* plasmids, while kanamycin (5 µg mL^−1^) and streptomycin (5 µg mL^−1^) were used for the expression analysis of toxin antitoxin genes in *A. citrulli*.

### Toxicity and antitoxicity assay

The full-length *Aave_1720*, *Aave_1719*, and *Aave_1720-Aave_1719* sequences, as well as the various altered versions of the *Aave_1720* and *Aave_1719* genes, were amplified by PCR using the sequence-specific primers listed in Table S5. After purification, the PCR products were cloned into the pBAD vector and transformed into *E. coli* Top10 competent cells. Single colonies were then isolated and inoculated in LB broth containing 50 µg mL^−1^ ampicillin and grown overnight before use in the toxicity spot assays. Sterile distilled water was added to the resulting cultures to produce serial dilutions, of which 3-µL aliquots were inoculated onto LBA plates containing 50 µg mL^−1^ ampicillin in the absence or presence of 0.2% (wt/vol) arabinose, which mediated the activity of the pBAD promoter. The plates were incubated at 37°C for 12 hours before being assessed for colony growth. Toxicity was also assessed in liquid culture in which overnight cultures were diluted to an OD_600_ of 0.1 in fresh LB containing 50 µg mL^−1^ ampicillin in the absence or presence of 0.2% (wt/vol) arabinose, and their absorbance at OD_600_ was monitored using the Bioscreen C System (Oy Growth Curves Ab Ltd., Helsinki, Finland) while growing at 37°C. The resulting data were used to construct growth curves in accordance with a method described previously (42).

### RNA isolation and RT-PCR

To extract total RNA, the AAC00-1 strain in the exponential phase was collected by centrifugation at 9500 × g and processed with the RNApure Bacteria Kit (CWBIO, Beijing, China). The RNA concentration and quality were determined using NanoDrop spectrophotometer (Thermo Fisher Scientific, MA, USA) and 1% TAE-agarose gels. To avoid genomic DNA contamination, the extracted RNA was incubated with 20 U DNase I for 30 min. 1 µg of total RNA was used for cDNA synthesis by the HiScript III 1st Strand cDNA Synthesis Kit (Vazyme, Nanjing, China). Equivalent cDNA were employed as PCR templates and using the same PCR system with KeyPo Master Mix (Vazyme, Nanjing, China), all primers used in this study were listed in Table S5. The reverse transcriptase (RT)-PCR products were separated on 1% TAE-agarose gels.

### Protein purification

The antitoxin gene *Aave_1719* was transferred to *E. coli* BL21 (DE3) (Tsingke, Beijing, China) and a pET28a vector that contained a GST-tag for expression of the Aave_1719 protein. During protein expression, the culture was initially grown overnight in LB broth supplemented with 50 µg mL^−1^ kanamycin, before 150 mL 1:100 subcultures were prepared in 250-mL flasks, and grown at 37°C with shaking at 200 rpm until reaching an OD_600_ of 0.6–0.8. Protein expression itself was induced by the addition of 1 mM isopropyl β-D-1-thiogalactopyranoside (IPTG). The cultures were subsequently incubated for a further 6 hours at 28°C with shaking at 150 rpm. However, the toxin gene *Aave_1720* was expressed in *E. coli* Top10 competent cells (Tsingke, Beijing, China) via the pBAD vector, which contained a His-tag. The strain was grown overnight with shaking in LB broth containing 50 µg mL^−1^ ampicillin and 0.2% (wt/vol) glucose and then inoculated at a 1:100 dilution into 150 mL LB broth supplemented with 50 µg mL^−1^ ampicillin and 0.2% (wt/vol) glucose for further cultivation. The culture was incubated at 37°C with shaking at 200 rpm until reaching an OD_600_ of 0.6–0.8, followed by centrifugation at 5,000 × *g* for 10 min at 4°C to collect the cell pellet. The cell pellet was resuspended in 150 mL LB broth supplemented with 50 µg mL^−1^ ampicillin and 0.2% (wt/vol) arabinose, and the induction of toxin protein Aave_1720 was performed at 28°C with shaking at 150 rpm for a further 6 hours. To purify the protein, the cells were pelleted by centrifugation at 5,000 × *g* for 10 min at 4°C and resuspended in 15 mL Lysis buffer (50 mM Tris pH 8, 150 mM NaCl, 1× protease inhibitor cocktail), with the buffer of the His-tagged proteins being supplemented with 10 mM imidazole. The cell walls were then disrupted by ultrasonication on ice, and the cell debris removed by centrifugation at 4°C. The resulting supernatants were loaded on Ni-NTA (BIORIGIN, Beijing, China) and glutathione resins (LABLEAD, Beijing, China) for the His-tagged and GST-tagged proteins, respectively. The bound protein was subsequently eluted with 1 mL elution buffer (50 mM Tris pH 8, 150 mM NaCl) containing 250 mM imidazole in the case of the His-tagged proteins and 10 mM reduced glutathione for the GST-tagged proteins. The resulting elution fractions were then concentrated before the proteins were harvested and transferred to storage buffer (50 mM Tris pH 8, 150 mM NaCl) using an Amicon Ultra Centrifugal Filter Unit (Merck Millipore, MA, USA). All samples were stored at −80°C until being required for further analysis.

### Site-directed mutagenesis

Individual point mutations were introduced to the *Aave_1720* and *Aave_1719* genes via inverse PCR as previously described (43), using two partially overlapping primers (Table S5), with the mutation site located within the overlapping section. The resulting PCR products were gel purified using a gel extraction kit (CWBIO, Beijing, China), before self-ligation using a seamless cloning kit (Vazyme, Nanjing, China), and transformation into *E. coli* Top10 and *E. coli* BL21 (DE3) competent cells (Tsingke, Beijing, China), respectively. All mutations were confirmed by DNA sequencing (Tsingke, Beijing, China).

### *In vitro* RNase assay

Total RNA from the *A. citrulli* model strain AAC00-1 was isolated using the RNApure Bacteria Kit (CWBIO, Beijing, China), while individual mRNAs of the *trpA*, *Aave_1720*, *Aave_1719*, *ompA*, and *atpE* genes were prepared by *in vitro* transcription using the T7 RiboMAX Express Large Scale RNA Production System (Promega, WI, USA). The RNase assay itself was conducted as described previously (44) with a few minor changes. The reaction mixture contained either 2 µg total RNA or 1 µg individual mRNA, 50 mM Tris-HCl (pH 8.0), 150 mM NaCl, and the appropriate quantity of purified Aave_1720 (8.87–67.5 pmol) or Aave_1719 (10–300 pmol) protein. The samples were then incubated at 30°C for 30 min before being mixed with loading dye and analyzed on either a 1.5% TAE-agarose gel or a 7% TBE-urea gel.

### Pull-down assay

The *Aave_1719* and *Aave_1720* genes were amplified by PCR and cloned into pET28a and pBAD vectors as described above, resulting in the pET28a-GST-*Aave_1719-*HA plasmid, which expressed the Aave_1719 protein with an N-terminus GST-tag and a C-terminus HA-tag, and pBAD-FLAG-*Aave_1720*-HIS, which expressed Aave_1720 with an N-terminus FLAG-tag and a C-terminus HIS-tag. The two plasmids were transformed into *Escherichia coli* BL21 (DE3) and *Escherichia coli* Top10 competent cells (Tsingke, Beijing, China), respectively. After purification, 10 µg of the relevant protein was applied to 70 µL of GST (LABLEAD, Beijing, China) or Ni-NTA agarose beads (BIORIGIN, Beijing, China). The sample volume was then adjusted to 1 mL with IP buffer (50 mM Tris, 150 mM NaCl, 1 mM EDTA, 1%EDTA) before being incubated at 4°C with orbital rotation. After 4 hours, the beads were washed with 1 mL IP buffer six times and finally resuspended in 1 × SDS-PAGE loading buffer (CWBIO, Beijing, China) and heated for 10 min at 100°C. The mixture was then centrifuged for 2 min before 10 µL of the supernatant was loaded on an SDS-PAGE gel, and western blots were performed using a primary antibody that recognized either the HA-tag (CWBIO, Beijing, China) or the FLAG-tag (CWBIO, Beijing, China).

### Yeast two-hybrid assay

The yeast two-hybrid experiments were carried out following the protocol of a previous study (45). Initially, the *Aave_1720* gene was amplified by PCR and cloned into the pGBKT7 vector in order to express the Aave_1720 protein fused to an N-terminus GAL4 DNA-binding domain (BD). Similarly, the *Aave_1719* gene was cloned into the pGADT7 vector so that the Aave_1719 protein was fused to an N-terminus GAL4 activation domain (AD). The two plasmids were then co-transformed into the Y2H *Saccharomyces cerevisiae* strain Y2HGold (Huayueyang, Beijing, China) and plated onto synthetic dropout (SD) media lacking leucine and tryptophan (SD/-Leu/-Trp). After incubation at 30°C for 48 hours, the resulting colonies were transferred to SD media that also lacked adenine and histidine (SD/-Ade/-His/-Leu/-Trp) and was coated with 100 µL 4 mg mL^−1^ 5-Bromo-4-chloro-3-indoxyl-α-D-galactopyranoside (X-α-gal, Huayueyang, Beijing, China). The inoculated plates were then incubated for a further 48 hours at 30°C, after which the growth and color change of the transformants was assessed.

### Construction of *Aave_1720* and *Aave_1720-Aave_1719* maker-less deletion mutants

The coding regions of the *Aave_1720* gene and entire *Aave_1720-Aave_1719* ORF were deleted from the wild-type *A. citrulli* model strain AAC00-1 using homologous recombination as described previously (46). Upstream and downstream fragments of the target genes were amplified by PCR using the primers listed in Table S5 and cloned into the suicide plasmid pk18mob*sacB*. The recombinant plasmids were then transformed into AAC00-1 by electroporation and selected on LBA supplemented with 50 µg mL^−1^ ampicillin and 50 µg mL^−1^ kanamycin. The transformant colonies produced were then cultured in LB broth in the absence of antibiotics before being plated on M9 medium supplemented with 10% sucrose. The ampicillin-resistant/kanamycin-sensitive deletion mutants were then selected and verified by PCR and DNA sequencing (Tsingke, Beijing, China).

### Biofilm formation assay

The wild-type *A. citrulli* strain AAC00-1, as well as the Δ*Aave_1720* and Δ*Aave_1720-Aave_1719* deletion mutants, was initially cultured overnight in LB liquid medium before being diluted in a ratio of 1:100 in fresh LB broth and transferred to a 24-well cell culture plate (Corning, NY, USA) in 1-mL aliquots. After 4 days of incubation at 30°C, the cultures themselves were discarded, and each well was washed three times with 1 mL water. The plates were then dried in an oven at 60°C for 30 min before 1 mL 1% crystal violet was added to each well. The plate was then incubated at room temperature for 30 min after which the wells were again washed three times with 1 mL water. The biofilm coating each well was then dissolved by the addition of 1 mL 95% ethanol, and its crystal violet content was assessed by measuring its absorbance at 590 nm using a U-T6A spectrophotometer (Yipuyiqi, Shanghai, China).

### Quantitative real-time PCR (qRT-PCR) analysis of gene expression

The expression levels of the *Aave_1720* and *Aave_1719* genes were assessed in the *A. citrulli* wild-type strain AAC00-1 under a range of stress-inducing conditions, which included low and high temperatures (4°C, 37°C, or 45°C), extreme pH (pH 4 and pH 10), exposure to ultraviolet (UV) light, redox stress (H_2_O_2_ or NaClO), metal ions (CuSO_4_), antibiotics (kanamycin and streptomycin), and osmotic stress (NaCl) as detailed in Table S3. Sample cultures were initially prepared in LB broth and incubated at 28°C for 12 hours until reaching the exponential growth phase. The cells were then harvested by centrifugation at 9,500 × *g* and washed twice in 0.85% NaCl before being resuspended in 0.85% NaCl amended with or subjected to the appropriated stress for a period of 30 min. The cells were then harvested and their total RNA was extracted using TRNzol Universal Reagent (Tiangen, Beijing, China) following the protocol of the manufacturer. The quality and quantity of the resulting RNA samples were determined using a NanoDrop Spectrophotometer (Thermo Fisher Scientific, MA, USA), before 2 mg of purified RNA was genomic DNA (gDNA) wiped and reverse transcription was carried out using the Hiscript III 1st Strand cDNA Synthesis Kit (Vazyme, Nanjing, China). Quantitative PCR was then performed using the ChamQ Universal SYBR qPCR Master Mix (Vazyme, Nanjing, China) and processed with the ABI QuantStudio 6 Flex Real-Time PCR system (Thermo Fisher Scientific, MA, USA). Relative quantification (ΔC_t_) of the data was then performed using *trpA*, *recA*, and *ugpB* as the reference genes.

### NaClO survival assay

Overnight liquid cultures (OD_600_ = 0.5) of the wild-type *A. citrulli* strain AAC00-1, as well as the Δ*Aave_1720* and Δ*Aave_1720-Aave_1719* deletion mutants, were harvested by centrifugation and washed twice in 0.85% NaCl before finally being resuspended at a concentration of 1.0 × 10^8^ cells/mL in 21 µM NaClO. After incubation at 28°C for 30 min, serial dilutions of the cells were prepared in 0.85% NaCl and plated on LB media to calculate the survival rate. The plates were then incubated at 28°C for 2 days before the number of colonies was counted.

### Statistical analysis

All experiments were repeated three times and the data were analyzed using the GraphPad Prism 7.0 software. Statistical differences were determined using a one-way analysis of variance (ANOVA) and Tukey’s multiple range test (*P* < 0.05).

## RESULTS

### *Aave_1719* and *Aave_1720* constitute a co-transcribed TA system

**Fig 1:**
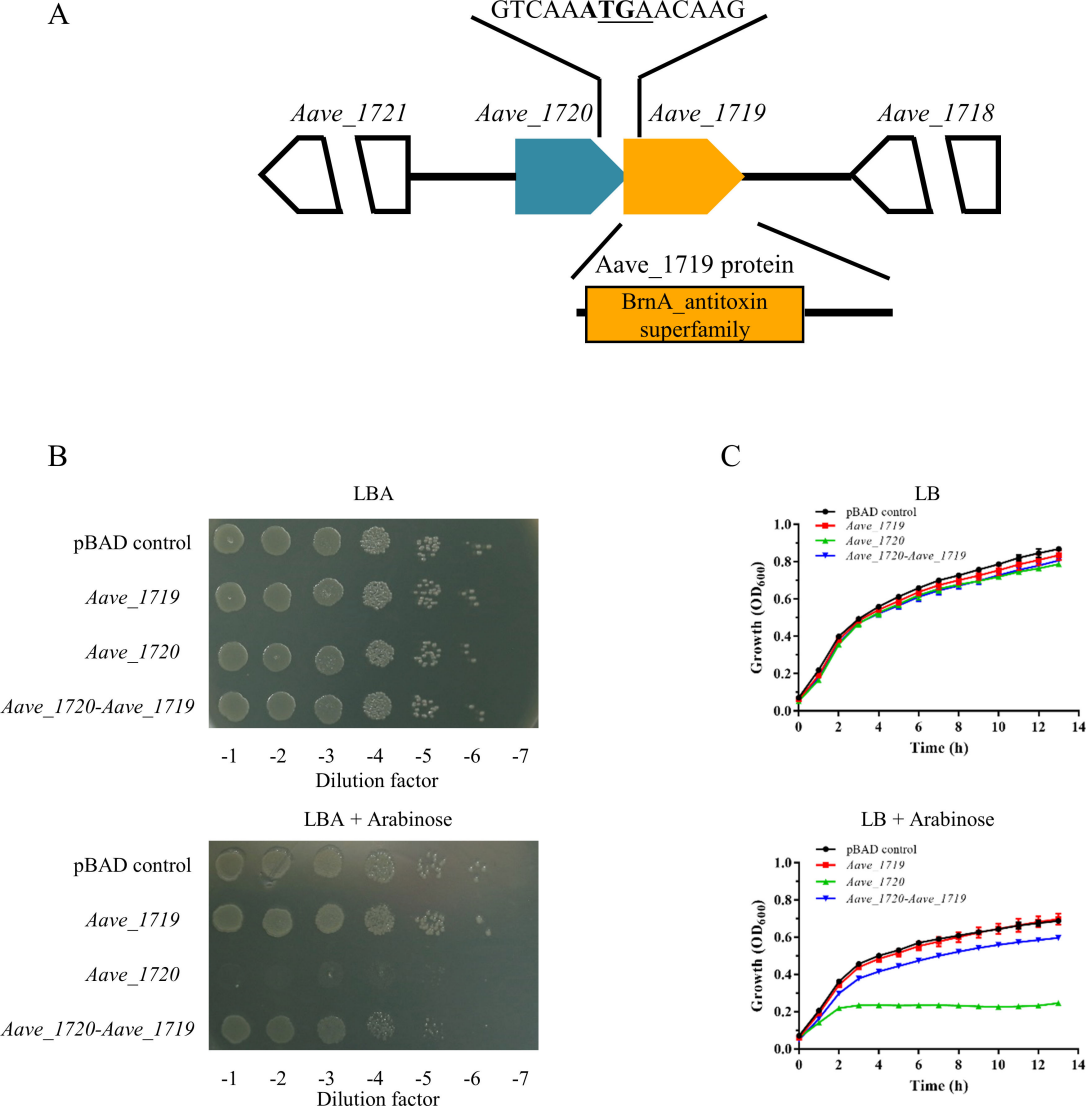
The *Aave_1720-Aave_1719* operon of *A. citrulli* encodes a toxin-antitoxin system. (**A**) Schematic of the *Aave_1720-Aave_1719* operon indicating the overlapping stop/start codon and the region of the operon with high homology to the BrnA antitoxin family. (**B**) Overnight cultures resulting from serial dilutions of *E. coli* transformed with pBAD-based plasmids containing an arabinose-inducible promoter driving either the toxin gene (*Aave_1719*), the antitoxin-gene (*Aave_1720*), or the entire operon (*Aave_1720-Aave_1719*). (**C**) Growth curves for the corresponding *E. coli* strains grown in LB broth in either the absence or presence of arabinose 0.2% (wt/vol). Data are representative of three independent experiments.

Searches of the type II toxin-antitoxin database (TADB 2.0) revealed the presence of a putative TA system in the *A. citrulli* genome (GenBank CP000512.1), which was encoded by a single operon located within the chromosomal DNA (1879411–1880030) of the *A. citrulli* model strain, AAC00-1 (41). Detailed analysis of the DNA sequence indicated that the stop codon of the *Aave_1720* toxin gene overlapped with the start codon of the *Aave_1719* antitoxin gene and that therefore, the toxin gene was located upstream of the antitoxin gene rather than being downstream, which is more usual in other TA systems (Fig. 1A). The bioinformatic analysis also predicted that the toxin encoded by *Aave_1720* was 99 amino acid residues in length, while the antitoxin was slightly longer at 107.

Heterologous expression in *E. coli* confirmed that the two genes did indeed constitute a functional TA system, as expression of the *Aave_1720* toxin under the control of an arabinose-inducible promoter resulted in virtually no growth on LBA when arabinose was present (Fig. 1B). In contrast, no similar reduction of growth was noted when transformants also expressing the antitoxin gene were grown on media containing arabinose. However, it was noted that expression of the full operon resulted in a slight reduction of growth, indicating that in this case, the presence of the antitoxin was able to neutralize the effect of the toxin, but not completely. Similar results were also obtained in liquid culture (Fig. 1C).

**Fig 2:**
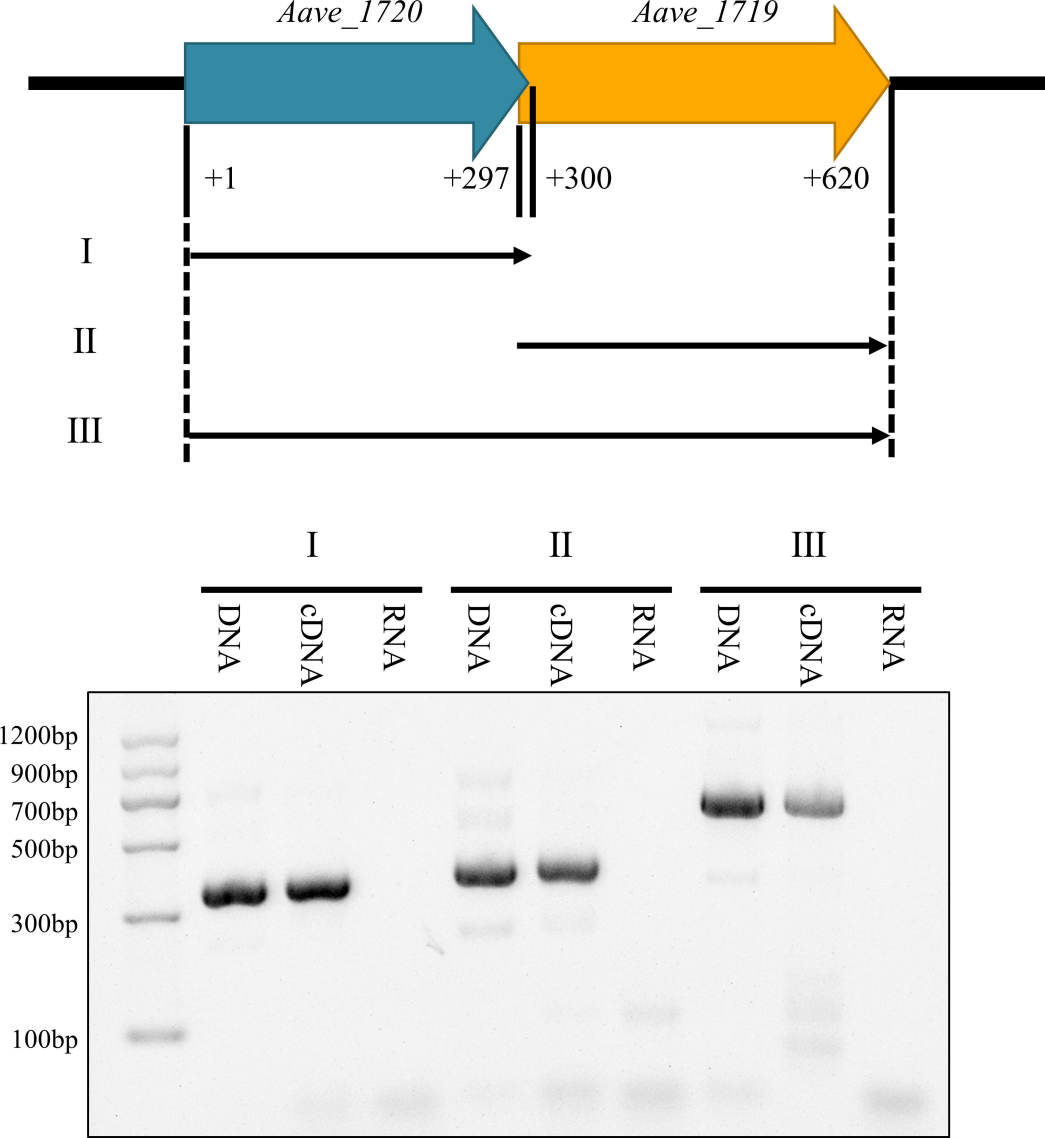
Evidence that *Aave_1720* and *Aave_1719* are co-transcribed. Three sets of primers were designed to amplify either *Aave_1720* (**I**), *Aave_1719* (II), or the entire *Aave_1720-Aave_1719* ORF (III) from the *A. citrulli* model strain AAC00-1. Comparison using total RNA, cDNA, or genomic DNA as templates for amplification demonstrated that PCR products of similar length were obtained from both the cDNA and genomic DNA, inferring that the two genes were co-transcribed as a single mRNA, while the differences in the relative amounts of product indicate that transcription of the individual genes is also possible.

The overlapping of the *Aave_1720* stop codon and the *Aave_1719* start codon indicated that the two genes might be co-transcribed. This was confirmed using RT-PCR with primers designed to the beginning of the *Aave_1720* gene and the end of the *Aave_1719* gene, which resulted in the amplification of a single product from the cDNA sample that was similar in length (620 bp) to the product obtained from the genomic DNA control (Fig. 2).

### Aave_1720 has ribonuclease activity

**Fig 3:**
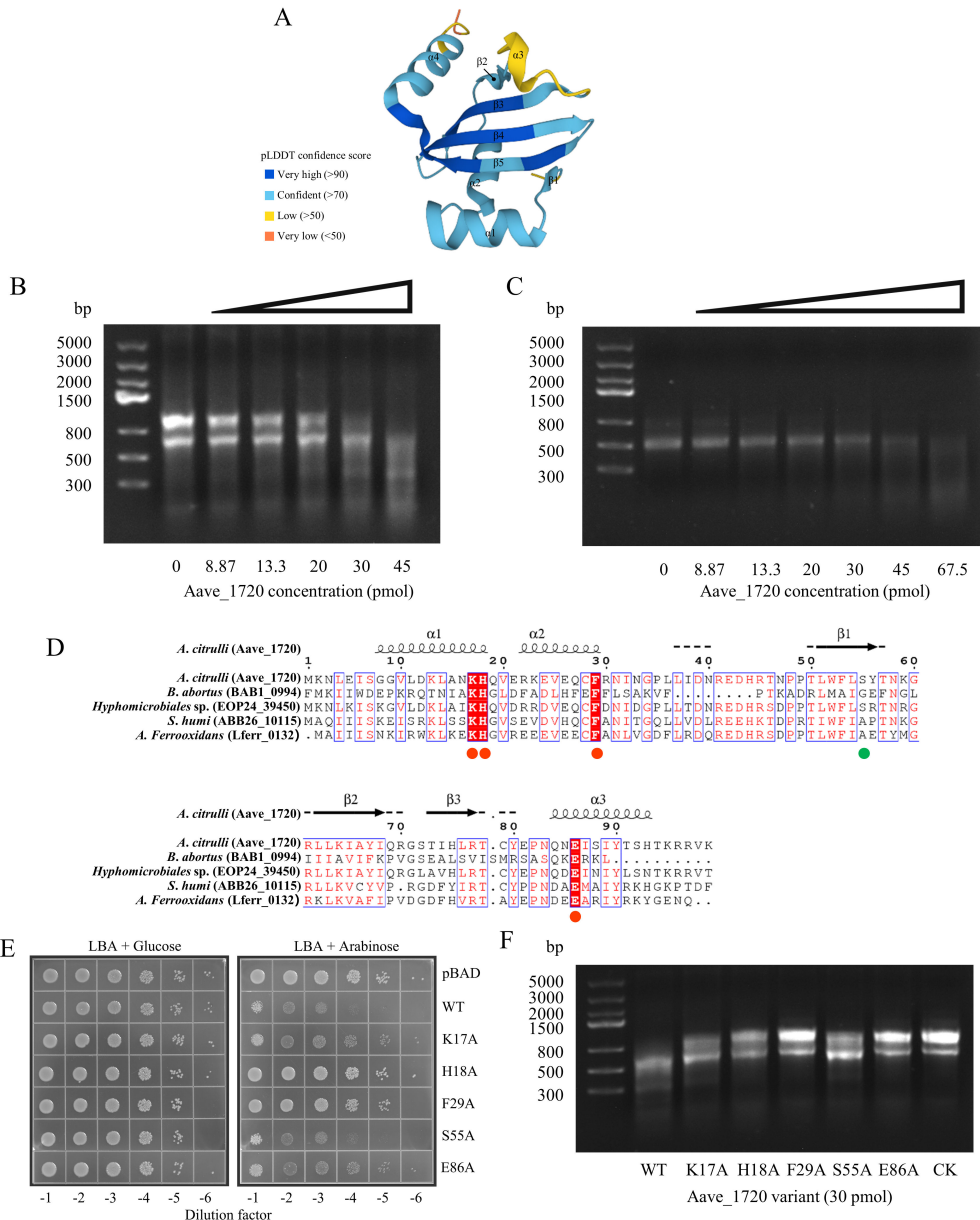
Four conserved residues in Aave_1720 essential for ribonuclease activity and toxicity. (**A**) Schematic showing the 3D structure of the Aave_1720 toxin protein as predicted by AlphaFold. Different colors indicate the per-residue confidence score (pLDDT) ranging from 0 and 100. (**B and C**) Gel images showing the results of RNase assays in which the Aave_1720 toxin (8.87–45 pmol) was incubated with either 2 µg *A*. *citrulli* total RNA (**B**), or 1 µg *in vitro* synthesized mRNA corresponding to the *A. citrulli* housekeeping gene *trpA* (**C**) at 30°C for 30 min. (**D**) Multiple sequence alignment of the predicted amino acid sequence of the Aave_1720 protein in comparison to homologous proteins from other bacteria, including BAB1_0994 of *Brucella abortus*, EOP24_39450 of *Hyphomicrobiales bacterium*, ABB26_10115 of *Stenotrophomonas humi*, and Lferr_0132 of *Acidithiobacillus ferrooxidans*. The four red dots indicate conserved residues that might be associated with RNase activity, while the green dot indicates a non-conserved residue that was evaluated for comparison. The alignment also indicates regions associated with the α-helices and β-sheet elements identified by PSIPRED secondary structure prediction software, which differed slightly to those predicted by AlphaFold. (**E**) Toxicity assay evaluating the effect of the four conserved amino acids (K17A, H18A, F29A, and E86A), as well as one non-conserved residue (S55A) on the toxicity of Aave_1720. Overnight LBA cultures of *E. coli* transformants expressing the wild-type (WT) *Aave_1720* gene as well as five modified variants under the control of an arabinose-inducible promoter on LBA supplemented with either 0.2% (wt/vol) arabinose or with 0.2% (wt/vol) glucose. (**F**) Gel image showing the results of an RNase assay in which 2 µg of *A. citrulli* total RNA was incubated with the wild-type Aave_1720 toxin (30 pmol) and five modified Aave_1720 variants carrying substitutions in conserved amino acid residues (K17A, H18A, F29A, E86A, and S55A) at 30°C for 30 min.

The mechanism by which Aave_1720 exerts toxicity was initially investigated using a bioinformatic approach based on its protein structure, as predicted by AlphaFold2 (47). The per-residue confidence score for 84 of the 99 Aave_1720 residues was in excess of 70 indicating a high level of confidence in the 3D structure predicted by the software (Fig. 3A). The putative protein structure was then used to search the DALI server for structural homologs (48), which identified four proteins with Z-scores in excess of 6 (PDB ID: 3u97, 5i4q, 3hi2, and 6l7q), with the closest match corresponding to the ribonuclease BrnT (Table. S1), which is known to be the toxin component of a previously characterized TA system occurring in the bacterium *B. abortus*. Given the implied function, the study went on to assay an Aave_1720 fusion protein, which was expressed in *E. coli* and purified via its His-tag (Fig. S1A), for RNase activity. Incubation of the purified protein with either *A. citrulli* total RNA or *in vitro* transcribed mRNA of the *trpA* housekeeping gene resulted in concentration-dependent degradation of the sample RNA, which was easily visible after electrophoresis (Fig. 3B and C). Taken together, these results provide strong evidence that Aave_1720 should be considered a ribonuclease toxin similar to BrnT.

### Four conserved key amino acid residues affect the toxicity and RNase activity of Aave_1720

Further bioinformatic analysis using the predicted amino acid sequence of Aave_1720 as the query for a BLASTP search of the RefSeq database (non-redundant protein sequences) identified several homologous proteins from a range of other bacterial species. Multiple sequence alignment identified several highly conserved amino acid residues that might be associated with RNase activity (Fig. 3D). To investigate further, a series of *Aave_1720* variants containing substitutions for these key residues (K17A, H18A, F29A, and E86A) were generated by a PCR-based cloning strategy and expressed in *E. coli* under the control of an arabinose-inducible promoter. The resulting transformants were plated on LBA in the absence or presence of arabinose, along with a similar variant containing the substitution of a non-conserved residue (S55A), as well as the wild-type *Aave_1720* gene as negative controls. The results confirmed that the histidine located at residue 18 and the phenylalanine at residue 29 were critical for toxicity as the strains carrying the H18A and F29A substitutions exhibited little sign of reduced growth in the presence of arabinose. However, the strains carrying the K17A and E86A substitutions exhibited partially reduced growth, indicating that these residues were less critical to the toxicity of Aave_1720, while the S55A substitution and wild-type gene resulted in an almost complete loss of growth (Fig. 3E). These results were further investigated using an RNase assay, which showed almost no degradation of *A. citrulli* total RNA resulting from incubation with Aave_1720 carrying the F29A substitution (Fig. 3F; Fig. S2). Interestingly, the E86A substitution also resulted in a complete loss of ribonuclease activity even though it exhibited only a slight decrease in toxicity, while conversely, H18A resulted in only a partial reduction in RNase activity, but a complete loss of toxicity. Similarly, it was interesting to note that even though the S55A substitution didn’t affect toxicity, it did slightly reduce RNase activity. Meanwhile, the partial reduction in toxicity associated with the K17A substitution corresponded well with its partial reduction in RNase activity. These results show that although there is a correlation between the RNase activity and toxicity of Aave_1720, the substitution of specific amino acids did not have a uniform effect, and further research is required to discover why the degree of RNase activity was not always directly proportional to toxicity.

### Aave_1719 inhibits the ribonuclease activity of Aave_1720 by forming a TA complex

**Fig 4:**
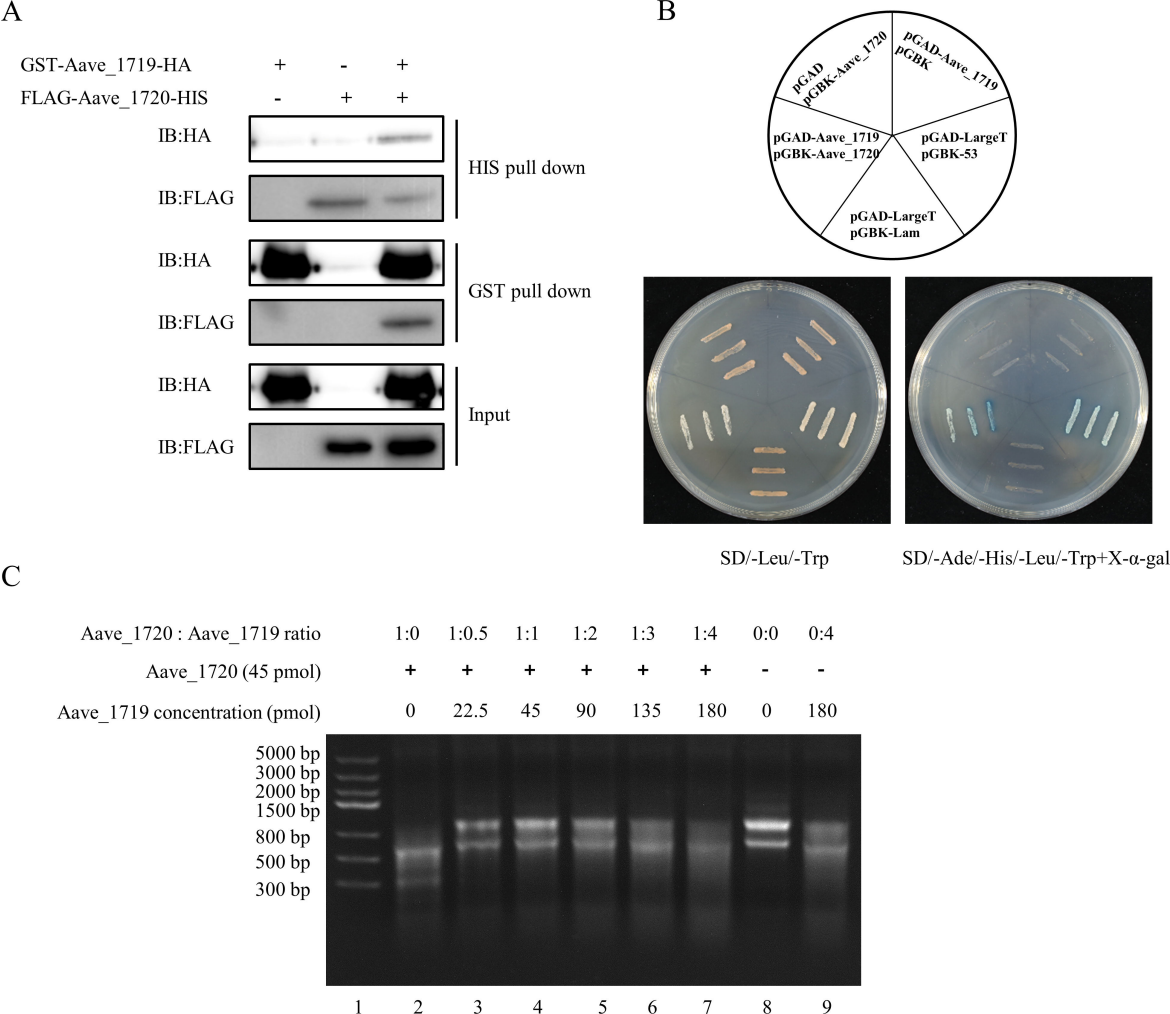
Aave_1719 inhibits the ribonuclease activity of Aave_1720 by the formation of a TA complex. (**A**) Pull-down assays confirm that the Aave_1720 toxin interacted with the Aave_1719 antitoxin via the use of FLAG/HIS and GST/HA fusion proteins. (**B**) Yeast two-hybrid assay with the *S. cerevisiae* strain Y2HGold co-transformed with pGAD and pGBK vectors carrying the *Aave_1719* antitoxin and *Aave_1720* toxin genes, respectively, as well as a positive control transformed with pGAD-LargeT and pGBK-53 and a negative control transformed with pGAD-LargeT and pGBK-Lam. (**C**) Gel image showing the results of an RNase assay in which 2 µg of *A. citrulli* total RNA was incubated with the Aave_1720 toxin (45 pmol) in the presence of various quantities of the Aave_1719 antitoxin (22.5–180 pmol), with a positive control where Aave_1719 was absent (lane 2) and a negative control where both proteins were absent (lane 8), as well as a second where just the toxin was absent (lane 9).

In type II TA systems, of which BrnTA is an example, the antitoxin is known to directly interact with the toxin to neutralize its toxicity. Given the similarity between Aave_1720 and BrnT, it was likely that the Aave_1719 antitoxin also functioned in such a way. This hypothesis was confirmed by pull-down assays in which an Aave_1720 fusion protein with an N-terminus FLAG-tag and a C-terminus HIS-tag was evaluated in conjunction with an Aave_1719 fusion protein with an N-terminus GST-tag and a C-terminus HA-tag. It was found that when the Ni-NTA agarose beads were used to pull down FLAG-Aave_1720-HIS as bait, the GST-Aave_1719-HA (prey) was also detected. Similarly, when GST beads were used with GST-Aave_1719-HA as bait, the FLAG-Aave_1720-HIS (prey) was also observed (Fig. 4A). This interaction between the Aave_1720 toxin and Aave_1719 antitoxin was further confirmed using a yeast two-hybrid assay. In this case, transformants carrying both the *Aave_1720* and *Aave_1719* genes were found to be capable of growth on a quadruple drop-out media similar to the pGAD-LargeT and pGBK-53 positive control, as well as to produce a blue color change from alpha-galactosidase activity, which confirmed the protein-protein interaction (Fig. 4B).

Having established that the Aave_1719 antitoxin interacted directly with the Aave_1720 toxin, an RNase assay was performed to investigate how this might affect the toxin’s activity. Incubation of Aave_1720 with *A. citrulli* total RNA and various concentrations of the Aave_1719 antitoxin revealed that this was indeed the case, with the RNase activity of the toxin being significantly reduced in the presence of low concentrations of the antitoxin, especially when the toxin and antitoxin were present in an equal ratio (Fig. 4C; Fig. S3). However, surprisingly, it was found that at higher concentrations of the antitoxin, RNase activity began to increase again and furthermore that RNA degradation occurred even when the toxin was absent. Taken together, these results indicate that although the antitoxin can bind to the toxin and neutralize its RNase activity, the unbound antitoxin itself possesses weak RNase activity.

### Aave_1719 has ribonuclease activity that preferentially targets *Aave_1720* mRNA

**Fig 5:**
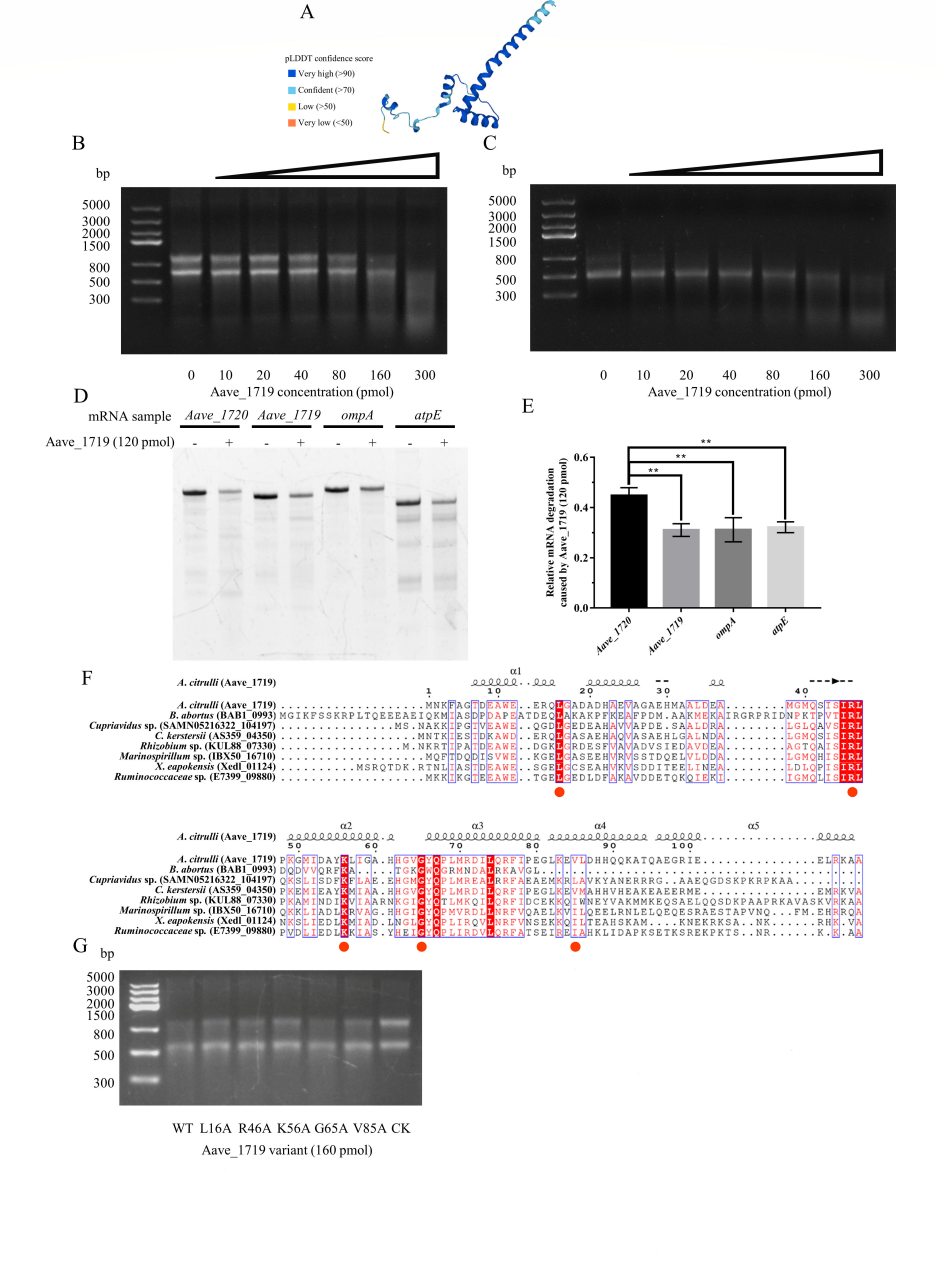
Aave_1719 exhibits ribonuclease activity that targets *Aave_1720* mRNA. (**A**) Schematic showing the 3D structure of the Aave_1719 antitoxin protein predicted by AlphaFold. Different colors represent the per-residue confidence score (pLDDT) ranging from 0 and 100. (**B and C**) Gel images showing the results of RNase assays in which the Aave_1719 antitoxin (10–300 pmol) was incubated with either 2 µg of *A. citrulli* total RNA (**B**), or 1 µg *in vitro* synthesized mRNA corresponding to the *A. citrulli* housekeeping gene *trpA* (**C**) at 30°C for 30 min. (**D**) Gel image showing the results of an RNase assay in which 1 µg *in vitro* synthesized mRNA corresponding to the *A. citrulli* genes *Aave_1720*, *Aave_1719*, *ompA*, and *atpE* were incubated in the absence or presence of the Aave_1719 antitoxin (120 pmol). (**E**) The fluorescence of the mRNA bands was converted to integrated density values to better compare the relative degradation of each of the four mRNA samples. Data represent three independent replicates, while asterisks indicate significant differences (*P* < 0.01) according to a one-way ANOVA test. (**F**) Multiple sequence alignment of Aave_1719 in comparison to homologous proteins from other bacteria including BAB1_0993 of *Brucella abortus*, SAMN05216322_104197 of *Cupriavidus* sp. *OV096*, AS359_04350 of *Comamonas kerstersii*, KUL88_07330 of *Rhizobium* sp., IBX50_16710 of *Marinospirillum* sp., Xedl_01124 of *Xenorhabdus eapokensis*, and E7399_09880 of *Ruminococcaceae bacterium*. The five red dots indicate conserved residues that might be associated with ribonuclease activity in Aave_1719. The alignment also indicates regions associated with α helices identified by PSIPRED secondary structure prediction software, which differed slightly to those predicted by AlphaFold. (**G**) Gel image showing the results of an RNase assay in which 2 µg total RNA from *A. citrulli* was incubated with the wild-type Aave_1719 antitoxin (160 pmol) and five modified Aave_1719 variants carrying substitutions in conserved amino acid residues (L16A, R46A, K56A, G65A, and V85A) at 30°C for 30 min.

Further investigation was conducted in order to more clearly understand the biological function of the Aave_1719 antitoxin beginning with a bioinformatic approach and the generation of a structural model (Fig. 5A) in AlphaFold2 (47). The model was again used to search the DALI server (48) in order to identify homologous proteins that might give further clues to its function. However, although the search revealed that the top four results were also antitoxins (PDB ID: 4me7, 6a6x, 6x0a, and 2bsq), it provided no evidence of any enzyme activity (Table S2). The study therefore went on to investigate the RNase activity of Aave_1719 directly using the same approached applied to the Aave_1720 toxin. The results confirmed that the purified Aave_1719 protein (Fig. S1B) also exhibited concentration-dependent RNase activity with both *A. citrulli* total RNA and the *trpA* mRNA. However, the activity of Aave_1719 was significantly lower than that of Aave_1720, with 160 pmol of the antitoxin being required to achieve a similar level of RNA degradation as 30 pmol of the toxin (Fig. 3B, C, 5B and C).

Although RNase activity has not previously been reported in the antitoxins of other type II TA systems, it has been observed in the type V TA system, in which targeted degradation of the toxin mRNA is the primary mechanism of neutralization (25). A further RNase assay was therefore conducted to investigate whether Aave_1719 also exhibited this behavior. In this case, the purified Aave_1719 was incubated together with four *in vitro* transcribed mRNA samples corresponding to the *Aave_1720*, *Aave_1719*, *ompA*, and *atpE* genes. The results showed that even though the Aave_1719 protein (120 pmol) could degrade all four mRNA samples, it did indeed appear to degrade the *Aave_1720* mRNA more efficiently (Fig. 5D and E). To validate the ability of Aave_1719 to degrade *Aave_1720* mRNA *in vivo*, we introduced the *Aave_1719* gene under the control of tetracycline-inducible promoter into AAC00-1. Total RNA was extracted at different time points after induction of Aave_1719 expression, and the levels of *Aave_1720* and *atpE* transcripts were detected by reverse transcriptase PCR. Compared to *atpE*, the level of *Aave_1720* transcript significantly decreased after induction of Aave_1719 expression (Fig. S4). In summary, both *in vitro* and *in vivo* experiments suggest that Aave_1719 possesses ribonuclease activity and prefers to degrade *Aave_1720* mRNA.

Although the search of the DALI server failed to identify any homologous proteins with RNase activity, a BLASTP analysis using the predicted amino acid sequence of the Aave_1719 protein identified several homologous protein sequences from other bacterial species. Multiple sequence alignment (Fig. 5F) identified several conserved amino acid residues that might be associated with RNase activity. However, a similar analysis as that performed for Aave_1720, in which Aave_1719 variants containing a range of amino acid substitutions (L16A, R46A, K56A, G65A, and V85A) were assessed, did not find any evidence that particular amino acids were critical for RNase activity. Indeed, all of the purified Aave_1719 variants (Fig. S1B) retained quite substantial levels of RNase activity, although four substitutions (L16A, R46A, K56A, and V85A) did seem to result in a slightly reduced activity (Fig. 5G).

### Effect of Aave_1720-Aave_1719 on biofilm formation and response to external stresses

**Fig 6:**
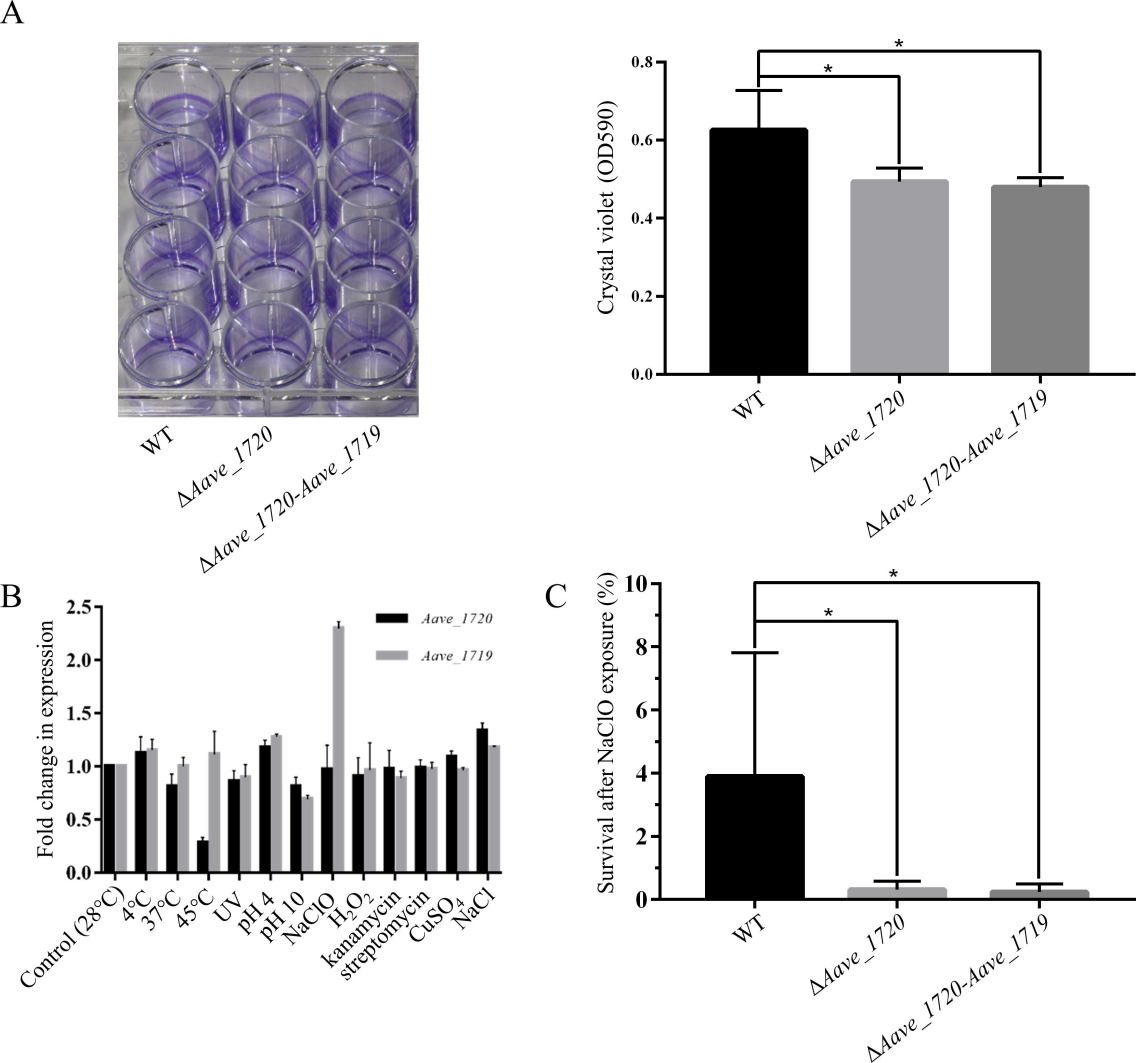
*Aave_1720-Aave_1719* contributes to biofilm formation and survival in response to sodium hypochlorite stress in *A. citrulli.* (**A**) Crystal violet staining of static *A. citrulli* cultures demonstrated a significant (*P* < 0.05) decrease in the accumulation of biofilm in deletion mutants lacking the capacity to produce the Aave_1720 toxin (Δ*Aave_1720* and Δ*Aave_1720-Aave_1719*) compared with the wild-type. Data represent three independent replicates, while asterisks indicate significant differences (*P* < 0.05) according to a one-way ANOVA test. (**B**) Quantitative PCR analysis showing the effect of various stress factors, including low and high temperatures (4°C, 37°C, and 45°C), extreme pH (pH 4 and pH 10), exposure to ultraviolet light, redox stress (H_2_O_2_, NaClO), metal ions (CuSO_4_), and osmotic stress (NaCl), as well as the antibiotics kanamycin and streptomycin, on the expression of both the *Aave_1720* toxin gene and the *Aave_1719* antitoxin gene in the *A. citrulli* model strain AAC00-1. Data represent the average of three biological replicates, with bars indicating 1 standard deviation. (**C**) Survival (%) of *A. citrulli* cells after exposure to NaClO (21 µM) for 30 min. Data represent the average of three independent replicates, with asterisks indicating significant differences (*P < 0.05*) according to a one-way ANOVA test.

Previous studies have shown that many TA systems play an important role in biofilm formation (15). The current study utilized homologous recombination to produced deletion mutants of the *Aave_1720* toxin gene and the entire *Aave_1720-Aave_1719* ORF in order to investigate whether there was any effect on biofilm formation using a crystal violet assay. The results revealed that biofilm formation was significantly reduced in both mutants compared with the wild-type strain (Fig. 6A). Furthermore, given that there was little difference between the Δ*Aave_1720* mutant and the Δ*Aave_1720-Aave_1719* mutant, it seems likely that the process in *A. citrulli* is primarily influenced by the Aave_1720 toxin itself.

It is well established that TA systems are frequently induced by stress as a mechanism to survive extreme environmental conditions (30, 49). Quantitative PCR was used to assess the type of stress that might cause activation of the Aave_1720-Aave_1719 TA system in *A. citrulli*. The results indicated that the expression levels of the *Aave_1720* and *Aave_1719* genes were slightly affected by most of the conditions tested, with only a significant reduction in the expression of *Aave_1720* occurring in response to heat shock (45°C) and a significant increase in expression of *Aave_1719* in response to sodium hypochlorite exposure (Fig. 6B). However, the study did go on to confirm that the Aave_1720-Aave_1719 TA system could have a significant effect on the survival of *A. citrulli*, as the Δ*Aave_1720* and Δ*Aave_1720-Aave_1719* deletion mutants were found to have a much lower rate of survival under stress induced by sodium hypochlorite exposure (Fig. 6C).

## DISCUSSION

**Fig 7:**
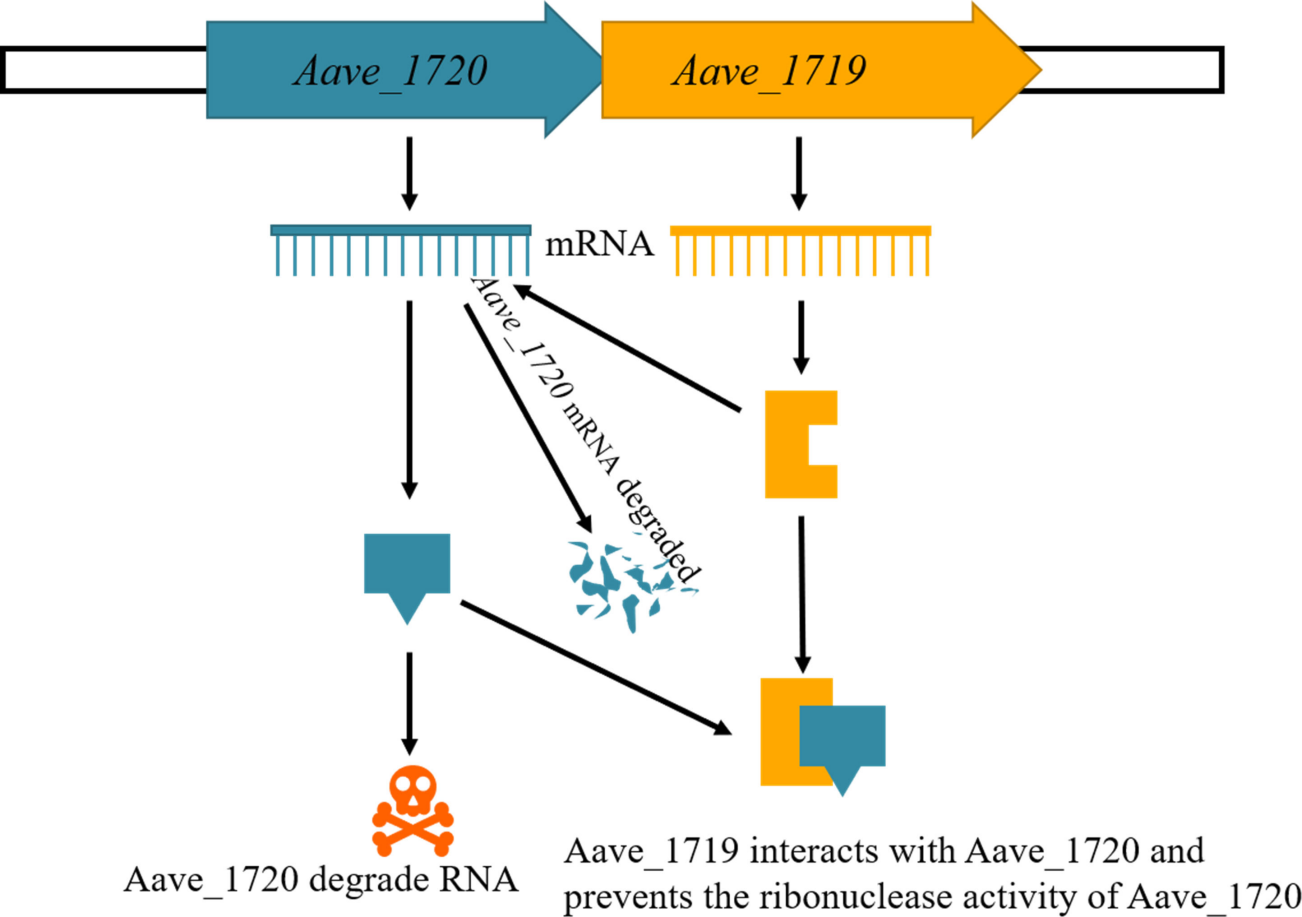
Proposed mechanism of the Aave_1720-Aave_1719 TA system. The RNase activity of the Aave_1720 toxin is inhibited by both the formation of an Aave_1720/Aave_1719 protein complex, as well as by the RNase activity of the Aave_1719 antitoxin, which exhibits preferential activity targeting the *Aave_1720* mRNA.

Toxin-antitoxin systems are a widespread phenomenon in prokaryotic organisms, and their function has been studied extensively. The process by which the antitoxin neutralizes the toxin has been used to classify TA systems into eight types, with most antitoxins appearing to function via a single mechanism (13). To date, there are only two exceptions to this, the HEPN-MNT system of *Shewanella oneidensis* and the DarTG system of *Mycobacterium tuberculosis*. In the first example, the MNT antitoxin inhibits the HEPN toxin not only by forming a complex with HEPN, which is characteristic of a type II TA system, but also by di-AMPylation of the toxin in process analogous to type VII TA systems (50). In the second example, the DarG antitoxin exhibits type II neutralization by directly binding the DarT toxin, but also via catalytic activity that counters that of the toxin, which is characteristic of a type IV TA system (24). The current study found compelling evidence that the novel Aave_1720-Aave_1719 TA system of *A. citrulli* also employs two mechanisms of toxin neutralization. Similar to the other two examples, it appears that the Aave_1719 antitoxin functions primarily by binding directly to the toxin to form an inactive TA complex in the manner of a type II TA system but that it also had secondary RNase activity that preferentially targets the toxin mRNA for degradation, which is characteristic of type V TA systems, as summarized in Fig. 7.

The BrnTA TA system was first characterized in 2009 (51) and is now considered a typical example of type II TA systems (30). It was therefore interesting to note that when surveying the DALI database, the putative structure of the *A. citrulli* toxin Aave_1720, as predicted by AlphaFold2, was found to be most similar to the BrnT ribonuclease toxin of *B. abortus* (Z-score = 8.7). However, the predicted structure of Aave_1720 differed from BrnT in two key respects, having an additional α-helix at its C-terminus (α4) and a beta sheet structure (β2) separating its α2 and α3 helices, which were joined directly in BrnT (30). However, despite these structural differences, the two proteins appeared to share the same function, with both the cytotoxicity and RNase activity of Aave_1720 being confirmed experimentally. Further bioinformatic analysis using multiple sequence alignment of homologous protein sequences from other bacterial species indicated that several conserved residues might be associated with RNase activity. Site-directed mutagenesis introducing substitutions of these key amino acids in Aave_1720 confirmed the importance of three amino acids, Lys-17, His-18, and Glu-86 that had previously been found to impact RNase activity in the BrnT toxin (30). However, the current study also confirmed that substitution of the phenylalanine located at residue 29 with the amino acid alanine resulted in a complete loss of both toxicity and ribonuclease activity in Aave_1720, which has not previously been reported in BrnT.

It was particularly interesting to discover that, in addition to forming a TA complex with the toxin, the Aave_1719 antitoxin also exhibited RNase activity. Although a search on the Conserved Domain Database indicated that the predicted structure of the Aave_1719 protein contained a BrnA_antitoxin-like domain at its N terminus (Fig. 1A), there have been no previous reports that BrnA has similar RNase activity. However, it has been shown that BrnA can bind the promoter of the *BrnTA* operon to facilitate autoregulation of expression (30). Therefore, it is hypothesized that the antitoxin Aave_1719 may also bind the promoter of the *Aave_1720-Aave_1719* operon. But, the results of the electrophoretic mobility shift assay (EMSA) showed that the Aave_1719 antitoxin failed to bind to the 200 bp DNA sequence upstream of the *Aave_1720* start codon and the *Aave_1719* start codon (Fig. S6A and B), which were the two predicted promoters according to SoftBerry BPROM analysis (Fig. S5). Meanwhile, the complex formed by the antitoxin Aave_1719 and the toxin Aave_1720 also failed to bind to the two promoters (Fig. S6C and D). Therefore, it is suggested that the antitoxin Aave_1719 might not regulate the transcription level of the *Aave_1720-Aave_1719* operon. Despite this uncertainty, the subsequent RNase assay confirmed that Aave_1719 did target *Aave_1720* mRNA for preferential degradation, similar to the type V antitoxin GhoS, which acts as a sequence-specific ribonuclease in *E. coli* (25). Therefore, it can be inferred that there may be some sequence repeats on the *Aave_1720* sequence that are overrepresented and therefore render the *Aave_1720* mRNA particularly sensitive to degradation by the antitoxin Aave_1719. Through MEME suite analysis, we discovered a 20-bp sequence (Fig. S7) that repeats four times in the ORF of *Aave_1720*, and they are evenly distributed in the ORF of *Aave_1720*. However, this 20-bp sequence exists only 2,828 times in the 5,352,772-bp genome sequence, which is much lower than their frequency in the ORF sequence (300 bp) of *Aave_1720*. Therefore, we speculate that this 20-bp sequence may be a potential target for the antitoxin Aave_1719, which also provides a direction for further experimental validation.

Most previous reports of TA system operons indicate that the antitoxin gene is usually located upstream of the toxin gene, which is probably a safeguarding mechanism that ensures the suppression of the toxin until it is required (13). However, there are some notable exceptions in which the antitoxin is located downstream, including the following TA systems: HigBA of *P. vulgaris* (52), MqsRA of *P. putida* (53), and HicAB of *E. coli* (54) TA systems. The current study found that the Aave_1720-Aave_1719 TA system was also one of these exceptional cases and furthermore that both genes were co-transcribed, as it was possible to amplify the entire *Aave_1720-Aave_1719* operon from both genomic DNA and cDNA using primers designed to the beginning of the *Aave_1720* gene and the end of the *Aave_1719* gene (Fig. 2). However, it was also noted that when the genes were evaluated individually, there appeared to be a greater quantity of *Aave_1719* product resulting from the cDNA sample, compared with that obtained from the entire *Aave_1720-Aave_1719* operon (Fig. 2). These results suggest that in addition to co-transcription, the *Aave_1719* gene can be transcribed independently to ensure the correct ratio of antitoxin to toxin. Supporting evidence for this hypothesis came from the SoftBerry BPROM (55) analysis of the *Aave_1720-Aave_1719* operon, which predicted the presence of two promoters, one located upstream of the *Aave_1720* gene (1880039–1880068) and another at the end of the *Aave_1720* ORF (1879733–1879765), as shown in Fig. S5. To validate the promoter activity, the upstream 204-bp sequence of *Aave_1720* and the upstream 200-bp sequence of *Aave_1719* were linked with the *lacZ* gene for transformation and expression in *E. coli* (Fig. S8). It was found that the upstream 200-bp sequence of *Aave_1719* had a strong promoter activity, confirming that the expression of the *Aave_1719* antitoxin is indeed mediated by two promoters simultaneously, similar to the mechanism previously observed in the MqsRA system of *E. coli* (56).

The widespread occurrence of TA systems among different prokaryotic organisms is generally considered to be an adaptation to various kinds of environmental stress (15). Biofilms, which are comprised of a matrix of extracellular polymeric substance (EPS) and the bacteria that produce them, are known to help many species resist the effects of adverse conditions including UV radiation, exposure to antibiotics, and extremes of temperature or pH and have been shown to be influenced by some TA systems (57). The current study found that the deletion of either the *Aave_1720* toxin gene or the entire *Aave_1720-Aave_1719* ORF significantly reduced biofilm formation in *A. citrulli*, which is similar to previous reports of the MqsRA TA system in *Pseudomonas putida*, as well as RelBE in *Vibrio cholerae* and yefM-yoeB in *Streptococcus pneumoniae* (15). The expression of toxin-antitoxin operons has also been correlated with the stress response of many bacterial species (14). However, the current study, found that only heat shock (45°C) or exposure to sodium hypochlorite significantly altered transcription in *A. citrulli*, resulting in the downregulation of *Aave_1720* and the upregulation of *Aave_1719*, respectively. Further investigation demonstrated that the altered expression in response to sodium hypochlorite could be linked to survivorship in *A. citrulli*, as the wild-type strain tolerated the stress significantly better than either the Δ*Aave_1720* or Δ*Aave_1720-Aave_1719* deletion mutants. Taken together, these results suggest that the Aave_1720-Aave_1719 TA system might function in a manner similar to the type II TA system HipBA of *C. crescentus* (49), in which sodium hypochlorite stress leads to the breakdown of the Aave_1719 antitoxin by intracellular proteases and the accumulation of unbound Aave_1720 toxin promotes persister cell formation by degrading intracellular RNA. However, further studies are required to demonstrate this process occurs *in vivo*. In order to validate the conservation of the antitoxin Aave_1719 and toxin Aave_1720 during evolution, we conducted a BLASTP analysis using the protein sequences of Aave_1719 and Aave_1720 and found that this TA system has homologous proteins (percent identity > 30%) in other bacterial genera. Additionally, we observed that this TA system is highly conserved within the *Acidovorax* genus. However, we observed that the antitoxin and toxin of the same bacterial strain exhibit different evolutionary positions on the phylogenetic tree (Fig. S9). This indicates that the evolution of antitoxin and toxin in other bacterial is not synchronized, possibly due to the differential selective pressures acting on antitoxin and toxin during the evolutionary process. Nonetheless, the current study, which documents a novel double-ribonuclease TA system with the characteristics of both type II and type V systems, already represents a significant step in expanding our understanding of the role TA systems play in the lifecycle of the economically important plant pathogen *A. citrulli*.

## SUPPLEMENTAL MATERIAL

Supplemental figures and tables spectrum.02169-23-s0001.docx Fig. S1 to S9; Tables S1 to S5.

ASM does not own the copyrights to Supplemental Material that may be linked to, or accessed through, an article. The authors have granted ASM a non-exclusive, world-wide license to publish the Supplemental Material files. Please contact the corresponding author directly for reuse.
