# Supplementary material for: A novel double-ribonuclease toxin-antitoxin system linked to the stress response and survival of Acidovorax citrulli
Source: Microbiol Spectr. 2023 Oct 11;11(6):e02169-23. doi: 10.1128/spectrum.02169-23 (PMC10714953; doi:10.1128/spectrum.02169-23)
Supplement: Supplemental figures and tables — Fig. S1 to S9; Tables S1 to S5. [file spectrum.02169-23-s0001.docx]

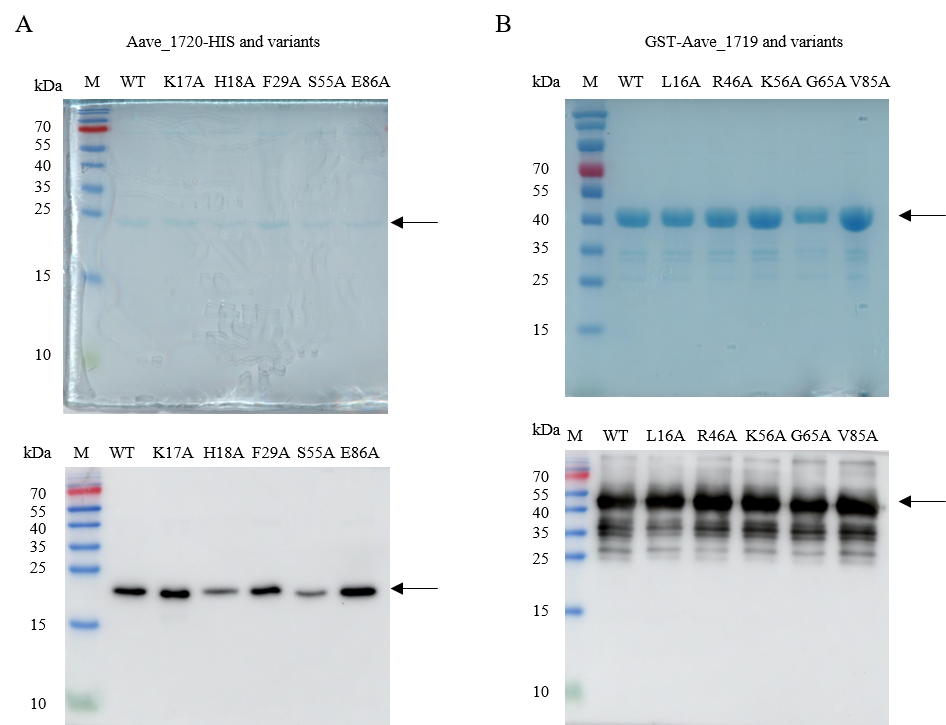


**Supplementary Fig. 1 Analysis of purified Aave_1720 and Aave_1719 proteins by SDS-PAGE and Western blot.** (A) Validation of purified wild-type (WT) Aave_1720-HIS and five modified Aave_1720 variants carrying substitutions in conserved amino acid residues (K17A, H18A, F29A, S55A, and E86A) by SDS-PAGE (up panel) and Western blot (lower panel). (B) Validation of purified wild-type (WT) GST-Aave_1719 and five modified Aave_1719 variants carrying substitutions in conserved amino acid residues (L16A, R46A, K56A, G65A, and V85A) by SDS-PAGE (up panel) and Western blot (lower panel).


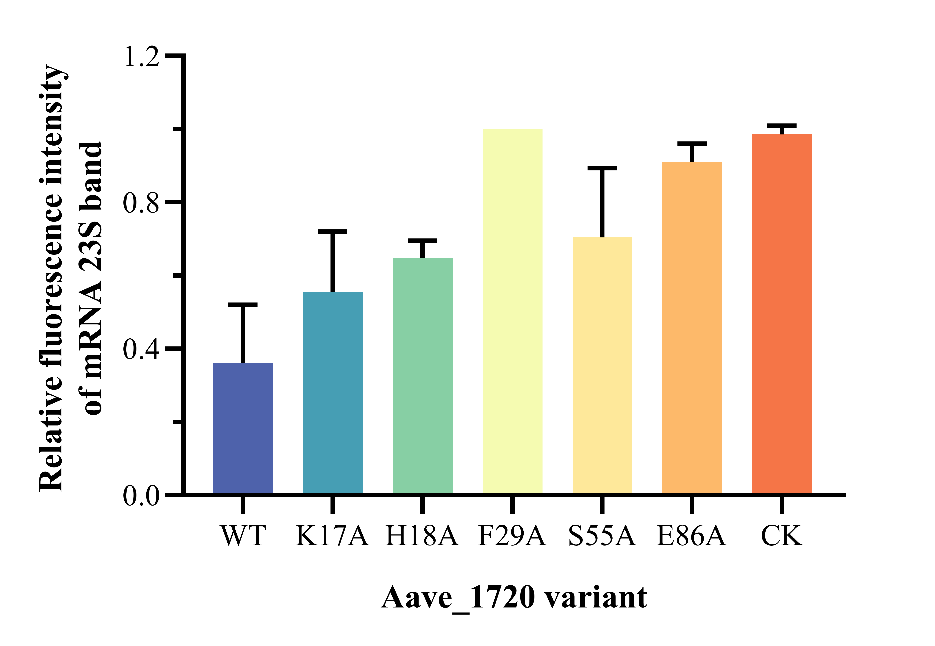


**Supplementary Fig. 2** The relative fluorescence intensity of mRNA 23S band after incubating 2 μg of *A. citrulli* total mRNA with Aave_1720 protein of wild-type (WT) or its mutant at five conserved amino acid residues (K17A, H18A, F29A, S55A, and E86A). Data represent three independent replicates.


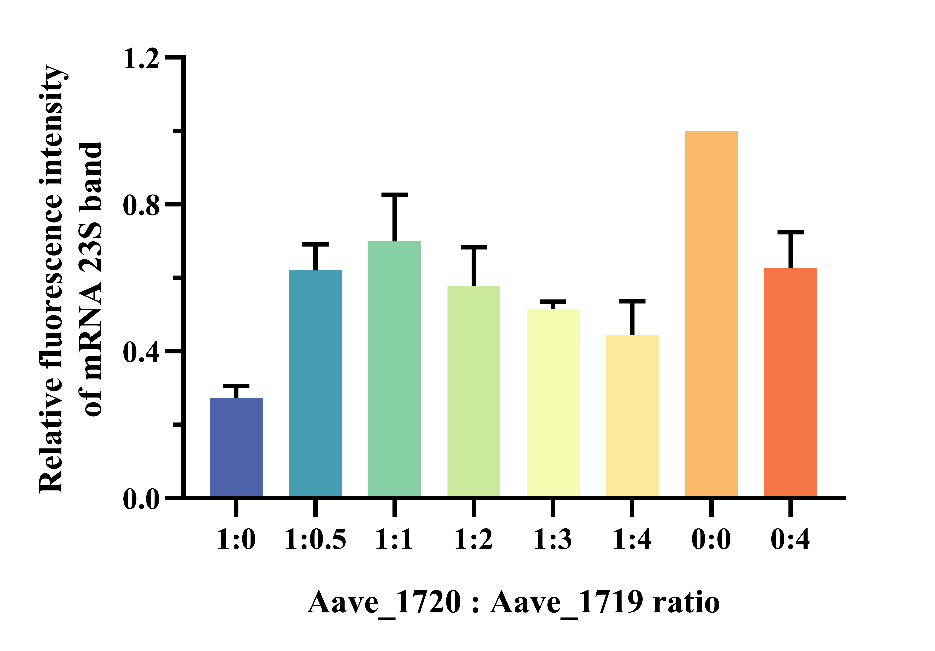


**Supplementary Fig. 3** The relative fluorescence intensity of mRNA 23S band after incubating 2 μg *A.citrulli* total mRNA with the Aave_1720 toxin (45 pmol) in the presence of various quantities of the Aave_1719 antitoxin (22.5-180 pmol). In this case, 1:0 represents the positive control with only Aave_1720 toxin, 0:0 represents the negative control with no proteins present, and 0:4 represents the negative control with only Aave_1719 antitoxin. Data represent three independent replicates.


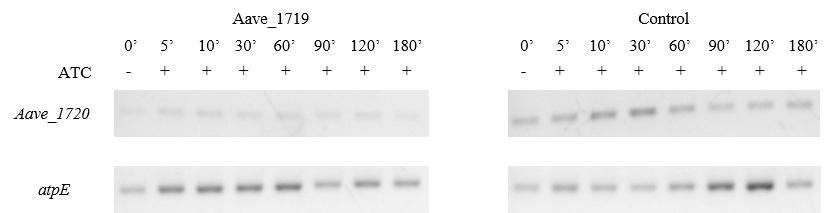


**Supplementary Fig. 4** The antitoxin Aave_1719 expression was induced by ATC (Anhydrotetracycline) in strain AAC00-1, and total RNA was extracted at indicated time points and the transcription levels of genes *Aave_1720* and *atpE* were evaluated using reverse transcriptase PCR and electrophoresis. The control was the transformation with empty vectors.


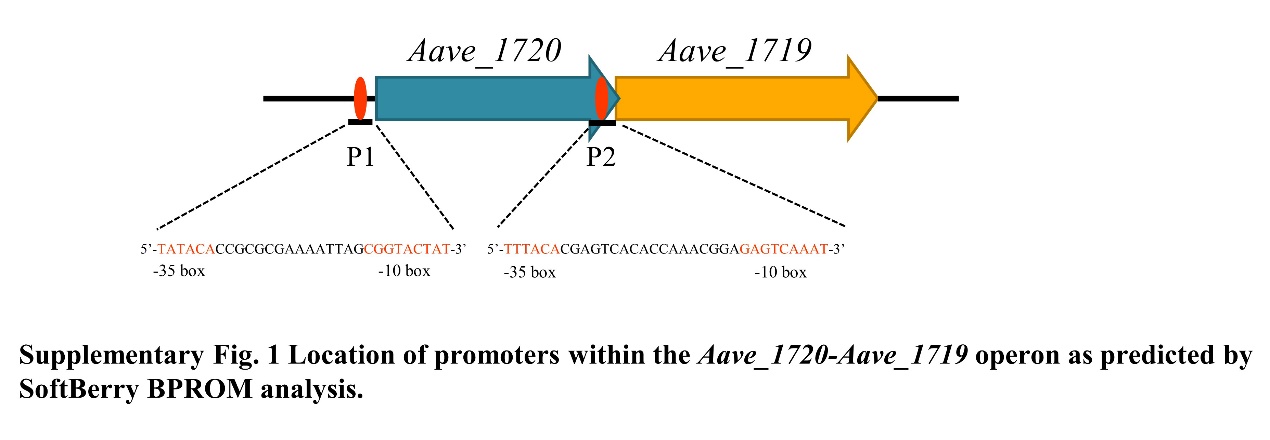


**Supplementary Fig. 5** Putative location of promoters within the *Aave_1720*-*Aave_1719* operon as predicted by SoftBerry BPROM analysis.


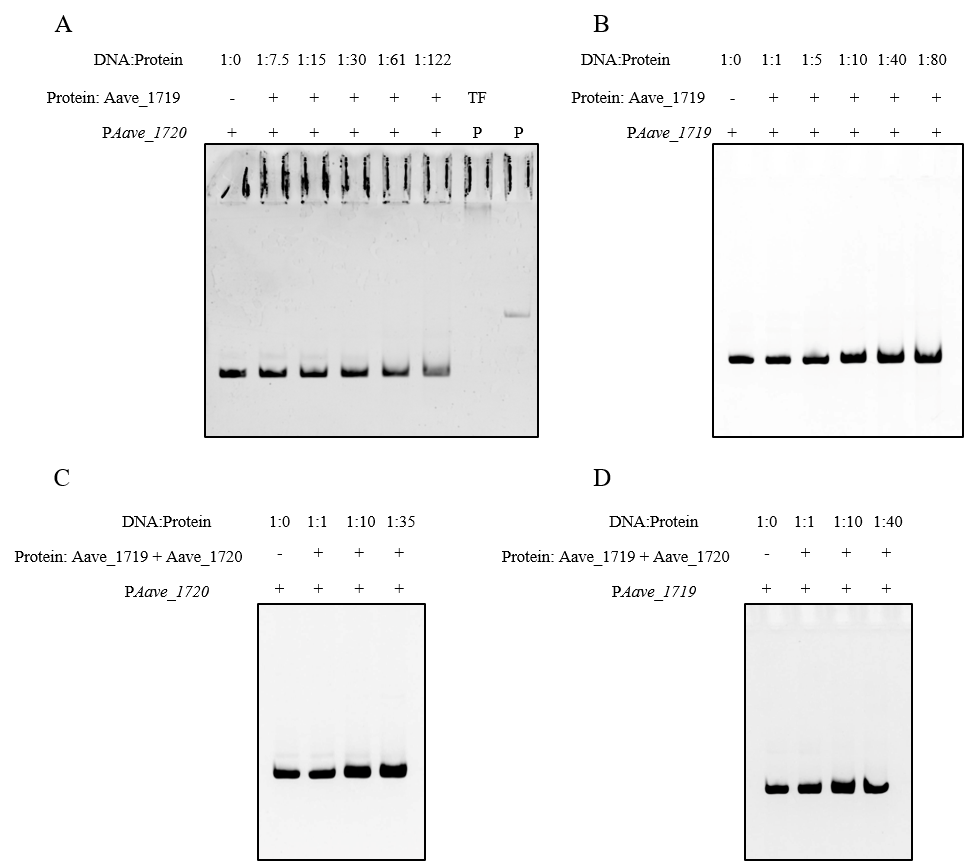


**Supplementary Fig. 6 The Aave_1719 antitoxin and the complex of Aave_1719 antitoxin and Aave_1720 toxin cannot bind to the predicted promoter of *Aave_1720-Aave_1719* operon.** (A and B) The Aave_1719 antitoxin cannot bind to the predicted promoters of the *Aave_1720* toxin gene (200 bp upstream of the *Aave_1720* start codon) (A), and the *Aave_1719* antitoxin gene (200 bp upstream of the *Aave_1719* start codon) (B). The transcription factor (TF) and promoter sequence (P) from *Clavibacter michiganensis* (unpublished data) were used as positive controls. (C and D) The complex of Aave_1719 antitoxin and Aave_1720 toxin cannot bind to the predicted promoters of the *Aave_1720* toxin gene (200 bp upstream of the *Aave_1720* start codon) (C), and the *Aave_1719* antitoxin gene (200 bp upstream of the *Aave_1719* start codon) (D).


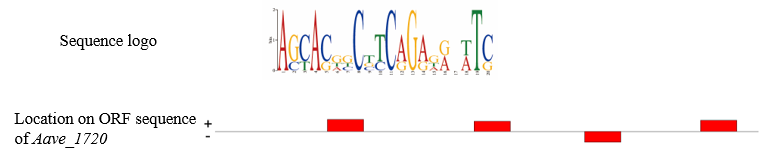


**Supplementary Fig. 7** Sequence logo representing the 20 bp overrepresented sequence present on the ORF sequence of *Aave_1720*, and the locations of the 20 bp sequence on the ORF sequence of *Aave_1720*. The red rectangle represents the 20 bp overrepresented sequence.


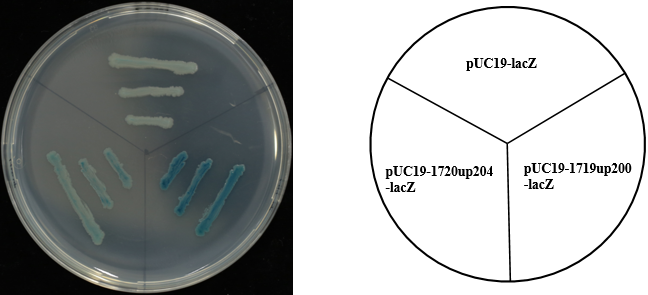


**Supplementary Fig. 8** *In vivo* validation of putative promoter activity. *E. coli* DH5α was transformed with pUC19 vector carrying the upstream 204 bp sequence of *Aave_1720* and *lacZ* gene (pUC19-1720up204-lacZ), and the upstream 200 bp sequence of *Aave_1719* and *lacZ* gene (pUC19-1719up200-lacZ), respectively. The negative control was the transformation with pUC19 vector carrying only the *lacZ* gene(pUC19-lacZ). The transformants were streaked on LB plates containing 100 μg ml^-1^ X-Gal, and the stronger activity of the promoter, the darker blue color of the colonies.


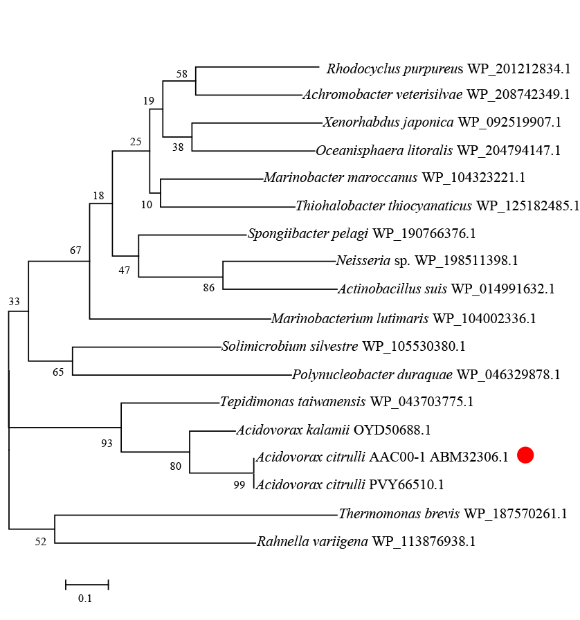


A


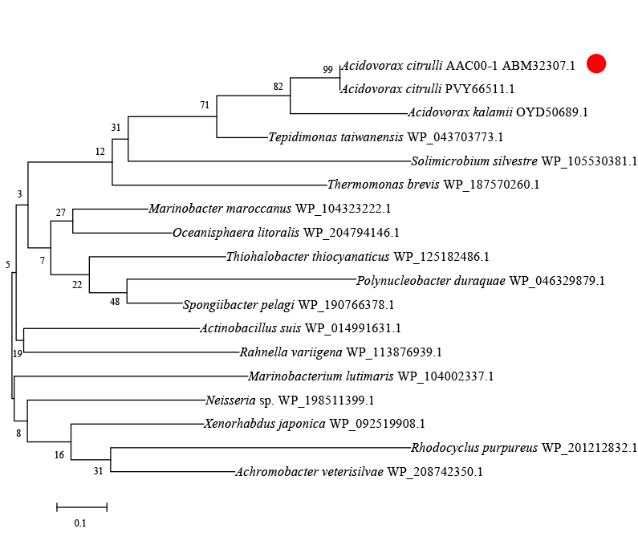


B

**Supplementary Fig. 9** The phylogenetic tree of antitoxin Aave_1719 and its homologous proteins (A), toxin Aave_1720 and its homologous proteins (B). Aave_1719 and Aave_1720 are marked by a red dot. The phylogenetic tree was constructed using the maximum-likelihood (ML) method by the software MEGA, and the phylogeny test adopted the Bootstrap method with 1000 replicates.

Supplementary Table S1. DALI results for Aave_1720.

| PDB ID | Z-SCORE | RMSD | LALI | PDB DESCRIPTION |
| --- | --- | --- | --- | --- |
| 3u97 | 8.7 | 2.1 | 74 | Ribonuclease toxin brnT |
| 5i4q | 7 | 2.2 | 72 | Contact-dependent inhibitor A |
| 3hi2 | 6.4 | 3 | 73 | HTH-type transcriptional regulator mqsa(ygit) |
| 6l7q | 6 | 3 | 71 | Hypothetical protein |
| 5mje | 5.7 | 3.1 | 71 | Cytotoxic translational repressor of toxin-antitoxin |
| 6n90 | 5.5 | 3.3 | 69 | Uncharacterized protein |
| 3g5o | 5.4 | 2.8 | 69 | Uncharacterized protein RV2865 |
| 1wmi | 5.4 | 3.6 | 72 | Hypothetical protein PHS013 |
| 3ao9 | 5.3 | 2.8 | 69 | Colicin-E5 |
| 5znm | 5.3 | 2.7 | 73 | Colicin-D |
| 2kc8 | 5.2 | 3.1 | 69 | Toxin rele |
| 3bpq | 5.1 | 3.3 | 67 | Antitoxin relb3 |
| 4mmg | 4.9 | 3.2 | 69 | mRNA interferase yafq |
| 6h0a | 4.7 | 3.7 | 68 | Serum paraoxonase-1 by directed evolution |
| 6cp9 | 4.7 | 3 | 74 | Cdia |
| 4ltt | 4.7 | 3.2 | 68 | Uncharacterized protein, toxin |
| 5ceg | 4.6 | 3.2 | 72 | Addiction module antidote protein |
| 6d7y | 4.6 | 2.6 | 65 | Hemagglutinin |
| 3kxe | 4.6 | 3.3 | 69 | Toxin protein pare-1 |
| 7btu | 4.5 | 3 | 58 | Putative tRNA (adenine(37)-n6)-methyltransferase |
| 5cw7 | 4.3 | 3.3 | 67 | Paaa2 |
| 6kml | 4.1 | 3.6 | 65 | mRNA interferase toxin higb |

Supplementary Table S2. DALI results for Aave_1719.

| PDB ID | Z-SCORE | RMSD | LALI | PDB DESCRIPTION |
| --- | --- | --- | --- | --- |
| 4me7 | 6.6 | 3.8 | 68 | Antitoxin EndoAI |
| 6a6x | 5.5 | 5.7 | 65 | Antitoxin MazE7 |
| 2gpe | 5.4 | 2.5 | 48 | Bifunctional protein putA |
| 6x0a | 5.3 | 3 | 67 | plasmid stabilization system |
| 2bsq | 5.2 | 6.5 | 64 | Trafficking protein A |
| 6sbx | 5 | 3.4 | 54 | CdbA |
| 3veb | 5 | 12.7 | 92 | Macrodomain Ter protein |
| 6gts | 4.9 | 6.6 | 66 | DUF1778 domain-containing protein |
| 5xe3 | 4.7 | 6.2 | 63 | Probable antitoxin MazE4 |
| 6gu1 | 4.5 | 2 | 55 | Secreted RxLR effector peptide protein, putative |
| 2kel | 4.5 | 1.5 | 45 | Uncharacterized protein 56B |
| 7emf | 4.5 | 9.6 | 80 | Mediator of RNA polymerase II transcription subunit 14 |
| 3on0 | 4.4 | 7.4 | 59 | Protein traM |
| 6k2j | 4.3 | 10.9 | 76 | UPF0335 protein CCNA_03428 |
| 3qoq | 4.2 | 2.7 | 50 | Alginate and motility regulator Z |
| 1arq | 4.2 | 2.8 | 49 | Arc repressor |
| 2k9i | 4.2 | 3.8 | 53 | Uncharacterized protein ORF56 |
| 3h87 | 4.2 | 6.5 | 55 | Putative uncharacterized protein |
| 6kyt | 4.2 | 8.1 | 60 | Antitoxin MazE9 |
| 2yf2 | 4.1 | 1.9 | 52 | C4B binding protein |
| 1ea4 | 4.1 | 2.3 | 44 | Transcriptional repressor Copg |
| 5x3t | 4 | 4.4 | 55 | Antitoxin VapB26 |

Supplementary Table S3. Stress conditions evaluated in the current study

| **Type of stress** | **Stress** |
| --- | --- |
| Physical stress | Heat shock 37°C |
|  | Heat shock 45°C |
|  | Cold shock 4°C |
|  | Ultraviolet light |
| Extreme pH | Acidic conditions (pH 4) |
|  | Alkaline conditions (pH 10) |
| Redox stress | Sodium hypochlorite (1.3 mM NaClO） |
|  | Hydrogen peroxide (0.25% H_2_O_2_) |
| Antibiotic response | Kanamycin sulfate (5 µg ml^-1^) |
|  | Streptomycin sulfate (5 µg ml^-1^) |
| Metal ions | Copper sulfate (5 µM) |
| Salt and osmotic stress | Sodium chloride (170 mM) |

Supplementary Table S4. Bacterial strains and plasmids used in the current study

(Continued on next page)

| **Bacterial strains/Plasmids** | **Description** | **Source** |
| --- | --- | --- |
| *E. coli* strains |  |  |
| DH5α | F^-^ Φ80d*lacZ* *ΔM15* Δ(*lacZYA*-*argF*) U169 *deoR* *recA1* *endA1* *hsdR17* (rK^-^, mK^+^) *phoA* *supE44* λ^-^ *thi*-*1* *gyrA96* *relA1* | Tsingke |
| TOP10 | F^-^ mcrA Δ(*mrr*-*hsd*RMS-*mcr*BC) Φ80 *lacZ* Δ*M15* Δ*lac*X74 *rec*A1 *ara*Δ139 Δ(*ara*-*leu*)7697 *gal*U *gal*K *rps*L (Str^R^) *end*A1 *nup*G | Tsingke |
| BL21(DE3) | F*^-^ompT hsdS_B_(r_B_^-^m_B_^-^) gal dcm*(DE3) | Tsingke |
| *Acidovorax citrulli* strains |  |  |
| AAC00-1 | Ap^r^; Wild-type group II model strain | Walcott et al., 2000 |
| Δ*Aave_1720* | In frame deletion of *Aave_1720* gene | The current study |
| Δ*Aave_1720-Aave_1719* | In frame deletion of *Aave_1720-Aave_1719* ORF | The current study |
| Plasmids |  |  |
| pBAD | Amp^R^; araBAD promoter, L-arabinose inducible expression vector for *E. coli* | Thermo Fisher |
| pBAD-*Aave_1720* | pBAD vector containing full-length (300 bp) *Aave_1720* toxin gene | The current study |
| pBAD-*Aave_1719* | pBAD vector containing full-length (324 bp) *Aave_1719* antitoxin gene | The current study |
| pBAD-*Aave_1720*-*Aave_1719* | pBAD vector containing full-length (620 bp) *Aave_1720-Aave_1719* ORF | The current study |
| pBAD- *Aave_1720 _K17A_* | pBAD vector containing *Aave_1720* toxin gene with altered sequence: AA to GC at nucleotides 49-50 | The current study |
| pBAD- *Aave_1720 _H18A_* | pBAD vector containing *Aave_1720* toxin gene with altered sequence: CAT to GCG at nucleotides 52-54 | The current study |
| pBAD- *Aave_1720 _F29A_* | pBAD vector containing *Aave_1720* toxin gene with altered sequence: TT to GC at nucleotides 85-86 | The current study |
| pBAD- *Aave_1720 _S55A_* | pBAD vector containing *Aave_1720* toxin gene with altered sequence: AGC to GCG at nucleotides 163-164 | The current study |
| pBAD- *Aave_1720 _E86A_* | pBAD vector containing *Aave_1720* toxin gene with altered sequence: A to C at nucleotide 257 | The current study |
| pET28a | Km^R^; T7 promoter, IPTG inducible expression vector for *E. coli* | Novagen |
| pET28a-GST-*Aave_1719*-HA | pET28a vector expressing Aave_1719 fusion protein with N-terminus GST and C-terminus HA tags | The current study |
| pET28a-GST-*Aave_1719 _L16A_*-HA | pET28a vector containing *Aave_1719* antitoxin gene with altered sequence: CT to GC at nucleotides 46-47 | The current study |
| pET28a-GST-*Aave_1719 _R46A_*-HA | pET28a vector containing *Aave_1719* antitoxin gene with altered sequence: CGA to GCG at nucleotides 136-138 | The current study |
| pET28a-GST-*Aave_1719 _K56A_*-HA | pET28a vector containing *Aave_1719* antitoxin gene with altered sequence: AA to GC at nucleotides 166-167 | The current study |
| pET28a-GST-*Aave_1719 _G65A_*-HA | pET28a vector containing *Aave_1719* antitoxin gene with altered sequence G to C at nucleotide 194 | The current study |
| pET28a-GST-*Aave_1719 _V85A_*-HA | pET28a vector containing *Aave_1719* antitoxin gene with altered sequence: T to C at nucleotide 254 | The current study |
| pK18*mobsacB* | Km^R^; Suicide vector for generating in-frame deletion mutants | The current study |
| pK18*mobsacB*-*Aave_1720* | pK18*mobsacB* containing homologous arms of the *Aave_1720* toxin gene | The current study |
| pK18*mobsacB*-*Aave_1720*-*Aave_1719* | pK18*mobsacB* containing homologous arms of the *Aave_1720*- *Aave_1719* ORF | The current study |

Supplementary Table S4. Bacterial strains and plasmids used in the current study (Continued)

Supplementary Table S5. Oligonucleotides used in the current study

| **Purpose/Name** | **Sequence (5’-3’)** | **Melting temperature (Tm)** |
| --- | --- | --- |
| Plasmid construction |  |  |
| pBAD-1720-F | TCATCATCATCATCATCATTCCATGGCTATGAAGAACTTAGAAATCTC | 47°C |
| pBAD-1720-R | ATATGGTACCAGCTGCAGATCTCGAGTCATTTGACTCTCCGTTTGG | 52°C |
| pBAD-1719-F | TCATCATCATCATCATCATTCCATGGCTATGAACAAGTTCGCAGGCAC | 58°C |
| pBAD-1719-R | ATATGGTACCAGCTGCAGATCTCGAGTCAGGCCGCCTTCCGCAGCT | 66°C |
| pBAD-FLAG-1720-F | ACAAGGACGATGACGATAAGATGAAGAACTTAGAAATCTC | 46°C |
| pBAD-FLAG-1720-R | TACCAGCTGCAGATCTCGAGTTTGACTCTCCGTTTGGTGT | 54°C |
| pET28a-1719-HA-R | AGCTCGAATTCGGATCCGCGTCAAGCGTAATCTGGAACGT | 54°C |
| pET28a-GST-1719-F | GATATACCATGGGCAGCAGCATGTCCCCTATACTAGGTTA | 49°C |
| BD-1720-F | TGATCTCAGAGGAGGACCTGCATATGATGAAGAACTTAGAAATCTC | 46°C |
| BD-1720-R | TGCGGCCGCTGCAGGTCGACGGATCCCTTTGACTCTCCGTTTGGTGT | 55°C |
| AD-1719-F | ACGACGTACCAGATTACGCTCATATGATGAACAAGTTCGCAGGCAC | 57°C |
| AD-1719-R | ATCTGCAGCTCGAGCTCGATGGATCCCGGCCGCCTTCCGCAGCTCCT | 68°C |
| PCR and DNA sequencing |  |  |
| pBAD-F | ATGCCATAGCATTTTTATCC | 51°C |
| pBAD-R | TCTGATTTAATCTGTATCAGG | 48°C |
| pET28a-F | TAATACGACTCACTATAGGG | 47°C |
| pET28a-R | GCTAGTTATTGCTCAGCGG | 55°C |
| M13F | TGTAAAACGACGGCCAGT | 48°C |
| M13R | CAGGAAACAGCTATGAC | 49°C |
| 1720-F | ATGAAGAACTTAGAAATCTC | 43°C |
| 1720-R | TCATTTGACTCTCCGTTTGG | 56°C |
| 1719-F | ATGAACAAGTTCGCAGGCAC | 58°C |
| 1719-R | TCAGGCCGCCTTCCGCAGCT | 72°C |

(Continued on next page)

Supplementary Table S5. Oligonucleotides used in the current study (Continued)

(Continued on next page)

| **Purpose/Name** | **Sequence (5’-3’)** | **Melting temperature (Tm)** |
| --- | --- | --- |
| Gene knockout |  |  |
| 1720up890-F | AATTCGAGCTCGGTACCCGGGGATCCAGCATCGTCTCGGCGTCG | 62°C |
| 1720up890-R | TTGTTCATTTCTTCATGGGATGCAATAGTA | 51°C |
| 1720down1000-F | TCCCATGAAGAAATGAACAAGTTCGCAGGC | 56°C |
| 1720down1000-R | GTAAAACGACGGCCAGTGCCAAGCTTGTCGAAGTGCGGCTTGCGAT | 62°C |
| 1720up500-F | AATTCGAGCTCGGTACCCGGGGATCCGCGTCGGAGTGCCAGTCGTT | 63°C |
| 1720up500-R | GGCATCAGGCCTTCATGGGATGCAATAGTAC | 52°C |
| 1719down500-F | TCCCATGAAGGCCTGATGCCGTACCTTGCC | 61°C |
| 1719down500-R | GTAAAACGACGGCCAGTGCCAAGCTTGGAGATTCTGCCGGCGCCTC | 63°C |
| *In vitro* transcription |  |  |
| T7-1720-F | GGATCCTAATACGACTCACTATAGGGACACCGCGCGAAAATTAGCGGT | 65°C |
| T7-1720-R | TCATTTGACTCTCCGTTTGGTGTGAC | 60°C |
| T7-1719-F | GGATCCTAATACGACTCACTATAGGGATGAACAAGTTCGCAGGCACTG | 60°C |
| T7-1719-R | TCAGGCCGCCTTCCGCAGCT | 66°C |
| T7-*trpA*-F | GGATCCTAATACGACTCACTATAGGGATGCGGGGCCATGCCGTCAA | 66°C |
| T7-*trpA*-R | CTACGTGTCGAGCGCCTTGC | 61°C |
| T7-*ompA*-F | GGATCCTAATACGACTCACTATAGGGATGAAGAAACTGAACAAAGTGGCGA | 58°C |
| T7-*ompA*-R | GTCGGCGGCGTAGGTCACCT | 65°C |
| T7-*atpE*-F | GGATCCTAATACGACTCACTATAGGGCTCAACCTTTCTTTCTTTTCAT | 51°C |
| T7-*atpE*-R | TCAGGCCAGGACGAAGGGGTTG | 64°C |
| Single amino acid substitution |  |  |
| 1720-K17A-F | TGGCAAACGCGCATCAAGTAGAGAGG | 43°C |
| 1720-K17A-R | ACTTGATGCGCGTTTGCCAATTTGTC | 45°C |
| 1720-H18A-F | CAAACAAGGCGCAAGTAGAGAGGAAA | 41°C |
| 1720-H18A-R | TCTACTTGCGCCTTGTTTGCCAATTT | 44°C |
| 1720-F29A-F | AGCAGTGCGCCAGGAACATCAATGGC | 47°C |
| 1720-F29A-R | ATGTTCCTGGCGCACTGCTCTACCTC | 49°C |

Supplementary Table S5. Oligonucleotides used in the current study (Continued)

| **Purpose/Name** | **Sequence (5’-3’)** | **Melting temperature (Tm)** |
| --- | --- | --- |
| 1720-S55A-F | GGTTTCTGGCGTATACCAACAAGGGC | 43°C |
| 1720-S55A-R | TTGGTATACGCCAGAAACCACAGCGT | 51°C |
| 1720-E86A-F | ACCAAAACGCGATCAGCATTTACACG | 45°C |
| 1720-E86A-R | ATGCTGATCGCGTTTTGGTTGGGCTC | 49°C |
| 1719-L16A-F | AGCGCCAAGCGGGCGCTGACGCCGAC | 59°C |
| 1719-L16A-R | TCAGCGCCCGCTTGGCGCTCCTCCCA | 54°C |
| 1719-R46A-F | TCTCGATCGCGCTGCCCAAAGGAATG | 48°C |
| 1719-R46A-R | TTGGGCAGCGCGATCGAGATGGACTG | 46°C |
| 1719-K56A-F | ACGCATACGCGCTGATCGGCGCTCAC | 53°C |
| 1719-K56A-R | CCGATCAGCGCGTATGCGTCGATCAT | 47°C |
| 1719-G65A-F | ATGGCGTCGCGTACCAGCCTCTGATG | 47°C |
| 1719-G65A-R | GGCTGGTACGCGACGCCATGGTGAGC | 53°C |
| 1719-V85A-F | TGAAGGAAGCGCTGGACCACCACCAG | 52°C |
| 1719-V85A-R | TGGTCCAGCGCTTCCTTCAACCCTTC | 45°C |
| qRT-PCR |  |  |
| 1719QF | TGCCCAAAGGAATGATCGAC | 60°C |
| 1719QR | ATGAAGCGTTGCAGGATGTC | 60°C |
| 1720QF | CTGGAGGCGTACTTGACAAA | 60°C |
| 1720QR | ATAGCTCAGAAACCACAGCG | 60°C |
| *recA*F | CGGTGGAGACGACCTGGAT | 60°C |
| *recA*R | AACAACGCCGCCAACAGC | 60°C |
| *trpA*F | GCCCAGGTGCTCGGCTATGT | 60°C |
| *trpA*R | CGCACTCTTCGGGAGGGTAA | 60°C |
| *ugpB*F | GGATTCGTATTCGTGCGTGGT | 60°C |
| *ugpB*R | CAAGAAGTGGGTGGACAGCG | 60°C |

Target nucleotides for substitution mutations are marked in red.
